# Discovery and Biosynthesis of Persiathiacins: Unusual Polyglycosylated Thiopeptides Active Against Multidrug Resistant Tuberculosis

**DOI:** 10.1021/acsinfecdis.4c00502

**Published:** 2024-08-27

**Authors:** Yousef Dashti, Fatemeh Mohammadipanah, Yu Zhang, Pietra M. Cerqueira Diaz, Anthony Vocat, Daniel Zabala, Christopher D. Fage, Isolda Romero-Canelon, Boyke Bunk, Cathrin Spröer, Lona M. Alkhalaf, Jörg Overmann, Stewart T. Cole, Gregory L. Challis

**Affiliations:** † Department of Chemistry, University of Warwick, Coventry CV4 7AL, U.K.; ‡ Sydney Infectious Diseases Institute, Faculty of Medicine and Health, University of Sydney, Sydney NSW 2015, Australia; § Pharmaceutical Biotechnology Lab, School of Biology and Center of Excellence in Phylogeny of Living Organisms, College of Science, University of Tehran, 14155-6455 Tehran, Iran; ∥ Global Health Institute, Ecole Polytechnique Fédérale de Lausanne, Station 19, 1015 Lausanne, Switzerland; ⊥ School of Pharmacy, Institute of Clinical Sciences, University of Birmingham, Birmingham B15 2TT, U.K.; # Leibniz-Institute DSMZ-German Collection of Microorganisms and Cell Cultures, 38124 Braunschweig, Germany; ¶ Technical University of Braunschweig, 38106 Braunschweig, Germany; ∇ German Centre of Infection Research (DZIF), Partner Site Hannover-Braunschweig, 38124 Braunschweig, Germany; ○ Warwick Integrative Synthetic Biology Centre, University of Warwick, Coventry CV4 7AL, U.K.; ⧫ Department of Biochemistry and Molecular Biology, Monash University, Clayton VIC 3168, Australia; †† ARC Centre of Excellence for Innovations in Peptide and Protein Science, Monash University, Clayton VIC 3168, Australia

**Keywords:** RiPP, antibiotic, 6-deoxysugar, cytochrome P450, glycosyl transferase

## Abstract

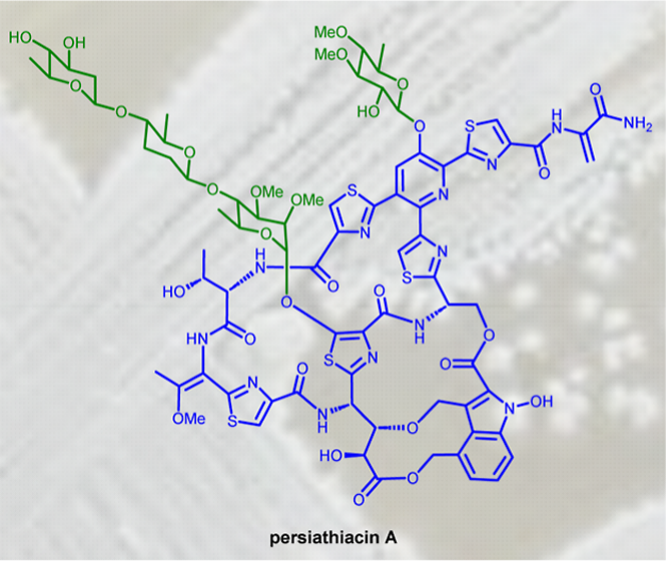

Thiopeptides are ribosomally biosynthesized and post-translationally modified peptides (RiPPs) that potently inhibit the growth of Gram-positive bacteria by targeting multiple steps in protein biosynthesis. The poor pharmacological properties of thiopeptides, particularly their low aqueous solubility, has hindered their development into clinically useful antibiotics. Antimicrobial activity screens of a library of Actinomycetota extracts led to discovery of the novel polyglycosylated thiopeptides persiathiacins A and B from *Actinokineospora* sp. UTMC 2448. Persiathiacin A is active against methicillin-resistant *Staphylococcus aureus* and several *Mycobacterium tuberculosis* strains, including drug-resistant and multidrug-resistant clinical isolates, and does not significantly affect the growth of ovarian cancer cells at concentrations up to 400 μM. Polyglycosylated thiopeptides are extremely rare and nothing is known about their biosynthesis. Sequencing and analysis of the *Actinokineospora* sp. UTMC 2448 genome enabled identification of the putative persiathiacin biosynthetic gene cluster (BGC). A cytochrome P450 encoded by this gene cluster catalyzes the hydroxylation of nosiheptide in vitro and in vivo, consistent with the proposal that the cluster directs persiathiacin biosynthesis. Several genes in the cluster encode homologues of enzymes known to catalyze the assembly and attachment of deoxysugars during the biosynthesis of other classes of glycosylated natural products. One of these encodes a glycosyl transferase that was shown to catalyze attachment of a D-glucose residue to nosiheptide in vitro. The discovery of the persiathiacins and their BGC thus provides the basis for the development of biosynthetic engineering approaches to the creation of novel (poly)glycosylated thiopeptide derivatives with enhanced pharmacological properties.

Over the past decade, *Mycobacterium tuberculosis* has caused up to 20 million deaths worldwide.^1^ In 2020, 1.5 million people died from tuberculosis, including 214,000 coinfected with HIV,^1^ and it was a leading infectious killer worldwide, second only to COVID-19.^1^ Currently, 6–12 month multidrug regimens are prescribed to treat *M. tuberculosis* infections. However, due to difficulties with dosing, side effects, and the emergence of multi and extensively drug-resistant strains, more effective antibiotics must be developed to combat this critical-priority pathogen.^2,3^

Thiopeptide antibiotics are ribosomally biosynthesized and post-translationally modified peptides (RiPPs). They are assembled from ribosomal peptide precursors via an extensive array of post-translational modifications catalyzed by a series of diverse enzymes.^4,5^ The precursor peptides consist of an N-terminal leader region that acts as a recognition motif for most of the post-translational modification enzymes and a C-terminal core region that is incorporated into the mature product(s).^5,6^ Common post-translational modifications of thiopeptides include azole formation via cyclodehydration/oxidation, dehydration of selected serine and threonine residues, and macrocyclization via [4 + 2] cycloaddition. Some thiopeptides are further modified via the introduction of additional macrocycles or the attachment of hydroxyl, methyl, indolyl, or quinaldyl substituents to the core peptide.^7−13^ Many thiopeptides possess potent activity against clinically relevant bacteria, in addition to antitumor and immunosuppressive properties.^14^ For instance, nosiheptide **1**, nocathiacin I **2**, and philipimycin **3**, which are representative of “series e” thiopeptides, are active against methicillin-resistant *Staphylococcus aureus* (MRSA) and/or clinical isolates of *M. tuberculosis* (Figure 1).^15−17^ Despite their promising bioactivity, thiopeptides have failed to reach the clinic due to poor aqueous solubility and gastrointestinal absorption. Several strategies, including biosynthetic pathway engineering, analogue total synthesis, and semisynthetic modification, have been applied to produce analogues with improved pharmacological properties.^18^

**Figure 1:**
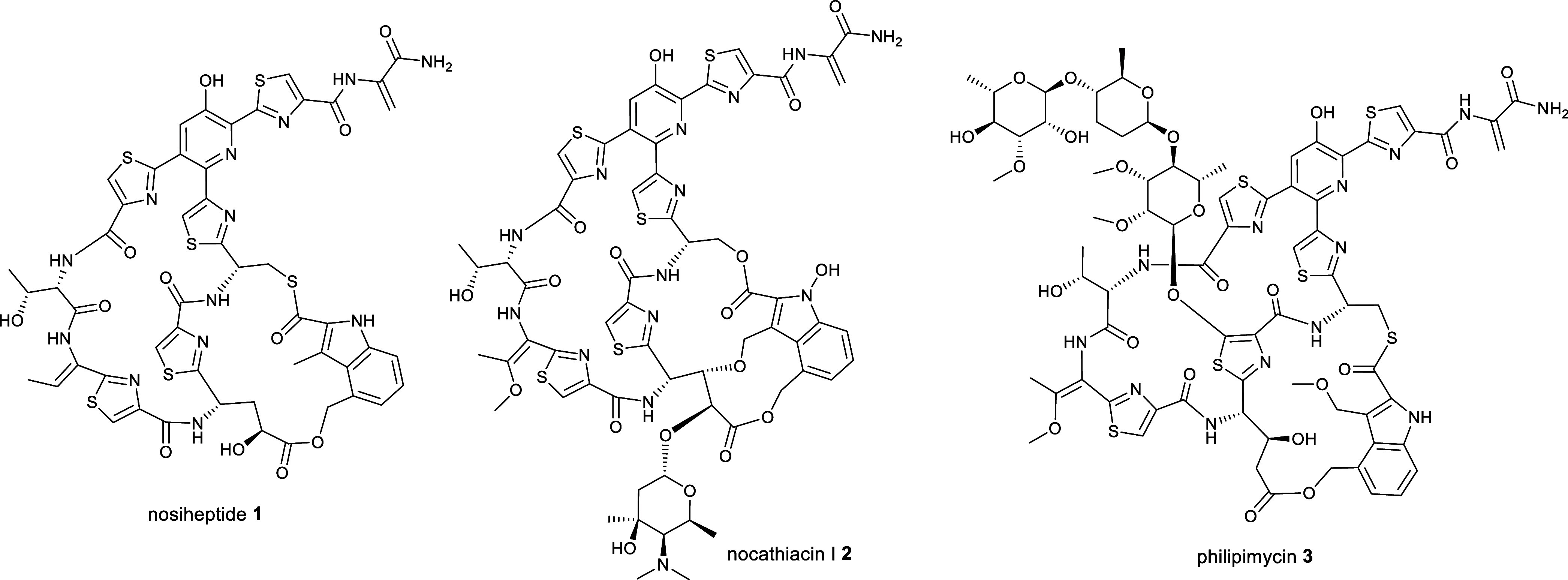
Structures of nosiheptide **1**, nocathiacin I **2**, and philipimycin **3**, which are examples of “series e” thiopeptide antibiotics.

Here, we report the discovery of the novel polyglycosylated thiopeptide antibiotics persiathiacins A and B, which are active against MRSA and drug-resistant *M. tuberculosis* clinical isolates. The persiathiacins are the first example of naturally occurring thiopeptides with a glycosylated hydroxypyridine and only the second example of antibiotics belonging to this class bearing a polyglycosylated hydroxythiazole. Glycosylation of the hydroxypyridine in nocathiacin has been reported to significantly improve aqueous solubility and, more generally, glycosylation is a widely used strategy for increasing the solubility and circulatory half-life of therapeutic peptides.^19−21^ Thus, the discovery of the persiathiacins and the gene cluster directing their biosynthesis provides new opportunities for the development of biosynthetic engineering strategies for the creation of novel (poly)glycosylated thiopeptides with improved pharmacological properties.

## Results and Discussion

### Isolation and Structure Elucidation of Persiathiacins A and B

**Figure 2:**
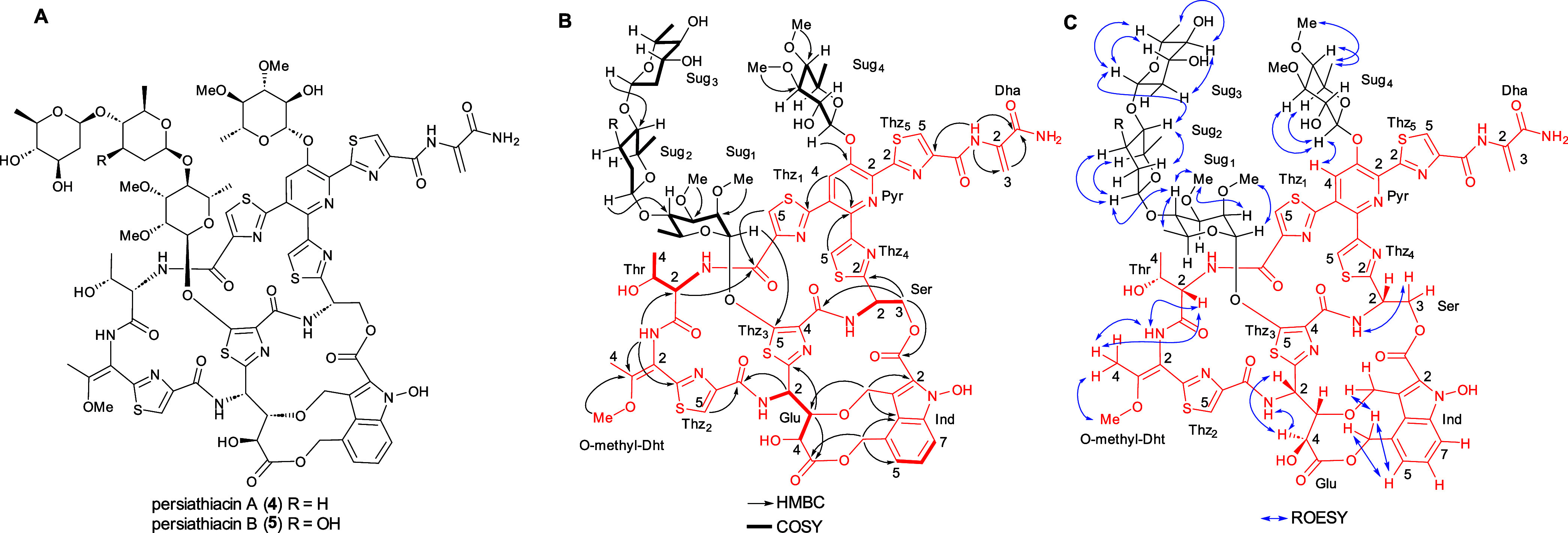
(A) Planar structures of persiathiacins A **4** and B **5**. (B) Summary of COSY and key HMBC correlations used to assign the planar structure of persiathiacins A **4** and B **5**. (C) Summary of key ROESY correlations observed for persiathiacins A **4** and B **5**.

During a search for novel natural products with activity against MRSA, Actinomycetota isolated from various locations in Iran were screened for antibiotic production. An ethyl acetate extract of *Actinokineospora* sp. UTMC 2448 was found to exhibit potent activity against MRSA. To identify the active metabolite(s), *Actinokineospora* sp. UTMC 2448 was cultured on solid ISP2 medium for 7 days, followed by ethyl acetate extraction and fractionation by semipreparative HPLC. A molecular formula of C_80_H_91_N_13_O_30_S_5_ was established from positive ion mode HR-ESI-MS and NMR data for the metabolite purified from the MRSA-active fraction. The planar structure of this compound, which we named persiathiacin A **4**, was elucidated using 1D and 2D NMR experiments (Figures 2 and S1, S3–S8, Table S1). Characteristic signals for amino acid α-protons at δ_H_ 4.21, 5.62, and 5.80, which correlated in HSQC spectra with α-carbon signals at δ_C_ 56.2, 48.6, and 48.7 and in COSY spectra with signals for exchangeable amide protons at δ_H_ 7.87, 7.89, and 8.24, indicated that the structure contains several amino acid residues. Four disubstituted thiazoles (including three bearing an acyl substituent at C4) and a tetrasubstituted pyridine were identified based on distinctive singlets due to protons attached to sp^2^-hybridized carbons in the ^1^H NMR spectrum, the chemical shifts of the signals for the directly connected carbon atoms, and HMBC correlations between these protons and neighboring carbons. A characteristic signal due to the sp^2^-hybridized methylene carbon of dehydroalanine (Dha) at δ_C_ 104.8, which showed HSQC correlations to two protons at δ_H_ 5.53 and 6.47, was also observed in the ^13^C NMR spectrum. This was further confirmed through ^2^*J* HMBC correlations between the methylene protons and the quaternary α-carbon of Dha at δ_C_ 133.5 and a ^3^*J* correlation to the carbonyl carbon at δ_C_ 166.5. Taken together, these data indicated that persiathiacin A has a thiopeptide core structure.

The following HMBC data showed that the core thiopeptide is very similar to that of the nocathiacins:^22^ a ^3^*J* correlation between the *O*-methyl protons (δ_H_ 3.78) and C3 (δ_C_ 158.7) of the *O*-methyl-dehydrothreonine (*O*-methyl-Dht) residue; ^3^*J* correlations between one of the methylene protons (δ_H_ 4.17) in the 3-alkoxymethyl substituent of the indole and C3 (δ_C_ 82.9) of the Glu residue, and between the C3 methine proton (δ_H_ 3.65) of the Glu residue and the methylene carbon (δ_C_ 65.9) of the indole 3-alkoxymethyl substituent; and a ^3^*J* correlation between an exchangeable hydroxyl group proton (δ_H_ 10.46) and C2 (δ_C_ 127.0) of the indole. The linkage of the 2-carboxyl group of the indole to the side chain of the serine residue was identified through the distinctive chemical shift of the signal due to the carbonyl carbon (δ_C_ 161.4), in comparison to those reported for nocathiacin I (161.1 ppm in dimethyl sulfoxide (DMSO)-*d*_6_) and nosiheptide (181.80 ppm in DMSO-*d*_6_), as well as the chemical shift of the signal for C3 of the Ser residue (δ_C_ 64.5), in comparison to the values reported for the corresponding carbon in nocathiacin I (63.3 ppm in DMSO-*d*_6_) and nosiheptide (29.5 ppm in DMSO-*d*_6_).^22,23^ A ROESY correlation between the protons attached to C4 and the NH of the 4-*O*-methyl-Dht residue established that it contains an *E*-configured double bond.

Studies of the solution conformation of nocathiacin I, indicate that the amide proton of the 4-*O*-methyl-Dht residue and the α-proton of the Thr residue, the amide proton and the γ-proton of the Glu residue, and the α- and γ-protons of the Glu residue, respectively, are in close spatial proximity.^24^ ROESY correlations between the corresponding protons in persiathiacin A are consistent with the Thr α-carbon and the stereocenters in the Glu residue having the same relative configurations as in nocathiacin I. Similarly, the splitting pattern of the signal for the less shielded of the diastereotopic C3 protons in the Ser residue, and a ROESY correlation between this proton and the Ser amide proton, indicate that the Ser α-carbon has the same relative configuration as in nocathiacin I. The only ambiguities are the relative stereochemistry of the β-carbon of the Thr residue and the absolute configuration of persiathiacin A. Given that persiathiacin A derives from a ribosomally biosynthesised precursor and there is a high degree of similarity between the persiathicin and nocathiacin biosynthetic gene clusters (BGCs) (see below), it seems highly likely that the Thr residue has the l, rather than the l-*allo*, d, or d-*allo*, configuration, as reported for nocathiacin I.^24^

In addition to the resonances assigned to the core thiopeptide, four distinctive signals at δ_C_ 100.4, 101.1, 101.9, and 102.2, assignable to anomeric carbons, that correlate in the HSQC spectrum with anomeric proton resonances at δ_H_ 5.18, 4.43, 5.41, and 4.61, respectively, were observed. These indicated that the thiopeptide core is decorated with four glycosyl residues (sugars 1–4). Four independent coupled proton spin systems, indicative of four distinct 6-deoxysugars, were identified by analysis of the COSY and HMBC spectra, and ^3^*J*_HH_ coupling constants (Figure 2 and Table S1). The HMBC spectrum was also used to identify the attachment site of each sugar and the locations of four *O*-methyl groups. A correlation between the anomeric proton of sugar 1 (δ_H_ 5.41) and C5 of thiazole 3 (δ_C_ 160.3) showed that sugar 1 is attached to C5 of thiazole 3. The positions of the *O*-methyl groups in sugar 1 were assigned based on 3-bond correlations between the protons of the methoxy groups and the carbons they are attached to. Thus, the protons in one of the *O*-methyl groups (δ_H_ 3.48) correlated with C2 (δ_C_ 76.0), while the protons in the other *O*-methyl group (δ_H_ 3.42) correlated with C3 (δ_C_ 80.1). HMBC correlations between the anomeric proton of sugar 2 (δ_H_ 4.61) and C4 of sugar 1 (δ_C_ 77.0), and between the C4 proton (δ_H_ 3.46) of sugar 1 and the anomeric carbon of sugar 2 (δ_C_ 102.2) established the connectivity between sugars 1 and 2. Similarly, HMBC correlations between the anomeric proton of sugar 3 (δ_H_ 4.43) and C4 of sugar 2 (δ_C_ 80.60) and between the C4 proton of sugar 2 (δ_H_ 3.08) and the anomeric carbon of sugar 3 (δ_C_ 101.1) showed that sugar 3 is attached to the C4 hydroxyl group of sugar 2. An HMBC correlation between the anomeric proton of sugar 4 (δ_H_ 5.18) and C3 of the pyridine (δ_C_ 149.0), in addition to a ROESY correlation between the C1 proton of sugar 4 and the C4 proton of the pyridine (δ_H_ 7.77), were consistent with the attachment of sugar 4 to the C3 hydroxyl group of the pyridine. Finally, HMBC correlations between the protons in one of the methoxy groups (δ_H_ 3.45) and C3 (δ_C_ 84.2) and the other methoxy group (δ_H_ 3.51) and C4 (δ_C_ 77.6) established the location of the *O*-methyl groups in sugar 4.

The signal due to the anomeric proton of sugar 1 is a broad singlet, suggesting it is the α-anomer, whereas the corresponding signals for sugars 2, 3, and 4 are doublets with ^3^*J*_HH_ values of 9.0, 10.0, and 7.5 Hz, respectively, indicative of β-anomeric linkages. Moreover, these coupling constants indicate that the protons attached to C2 of sugars 2, 3, and 4 are all axial. ROESY correlations between the C1 proton and the C2 methoxy group, the C2 and C4 protons and the C3 methoxy group, and the C4 and C6 protons in sugar 1 are consistent with this being a 2,3-di-*O*-methyl-α-l-rhamnose, as reported for the corresponding sugar in the philipimycins.^17^ Similarly, ROESY correlations between the protons attached to C1 and C5, and C2 and C4 in sugar 2 suggests they are all axial, consistent with this being d-amecitose, as also observed in the philipimycins. Sugar 3 is assigned as d-olivose, based on ROESY correlations between the protons attached to C1 and C3, C1 and C5, and C2 and C4. Finally, ^3^*J*_HH_ values of 7.5 and 10.0 Hz for the proton attached to C2, and ROESY correlations between the protons attached to C1 and C3, and C1 and C5, indicate that H1, H2, H3, and H5 in sugar 4 are all axial. Given that the persiathiacin BGC encodes only a single NDP-hexose 4-ketoreductase, which is required for the biosynthesis of d-amecitose and d-olivose (see below), both of which have an axial C4 proton, we propose that sugar 4 is a 3,4-di-*O*-methyl-6-deoxy-β-d-glucose residue.

In addition to persiathiacin A, a minor metabolite with a mass 16 Da greater than that of persiathiacin A was purified from the MRSA-active fractions of the *Actinokineospora* sp. UTMC 2448 extract. The molecular formula of this metabolite was deduced to be C_80_H_91_N_13_O_31_S_5_ from positive ion mode HR-ESI-MS and NMR spectroscopic data, indicating that it is a persiathiacin A derivative containing an additional oxygen atom. The NMR spectroscopic data for this minor compound, which we named persiathiacin B **5**, were almost identical to that for persiathiacin A, except for the proton and carbon resonances of sugar 2 (Figures S2 and S9–S14, Table S2). Detailed analysis of 1D and 2D NMR spectra indicated that the β-d-amicetose residue in persiathiacin A is replaced by β-d-olivose in persiathiacin B (Figure 2).

### Identification and Analysis of the Putative Persiathiacin BGC

**Figure 3:**
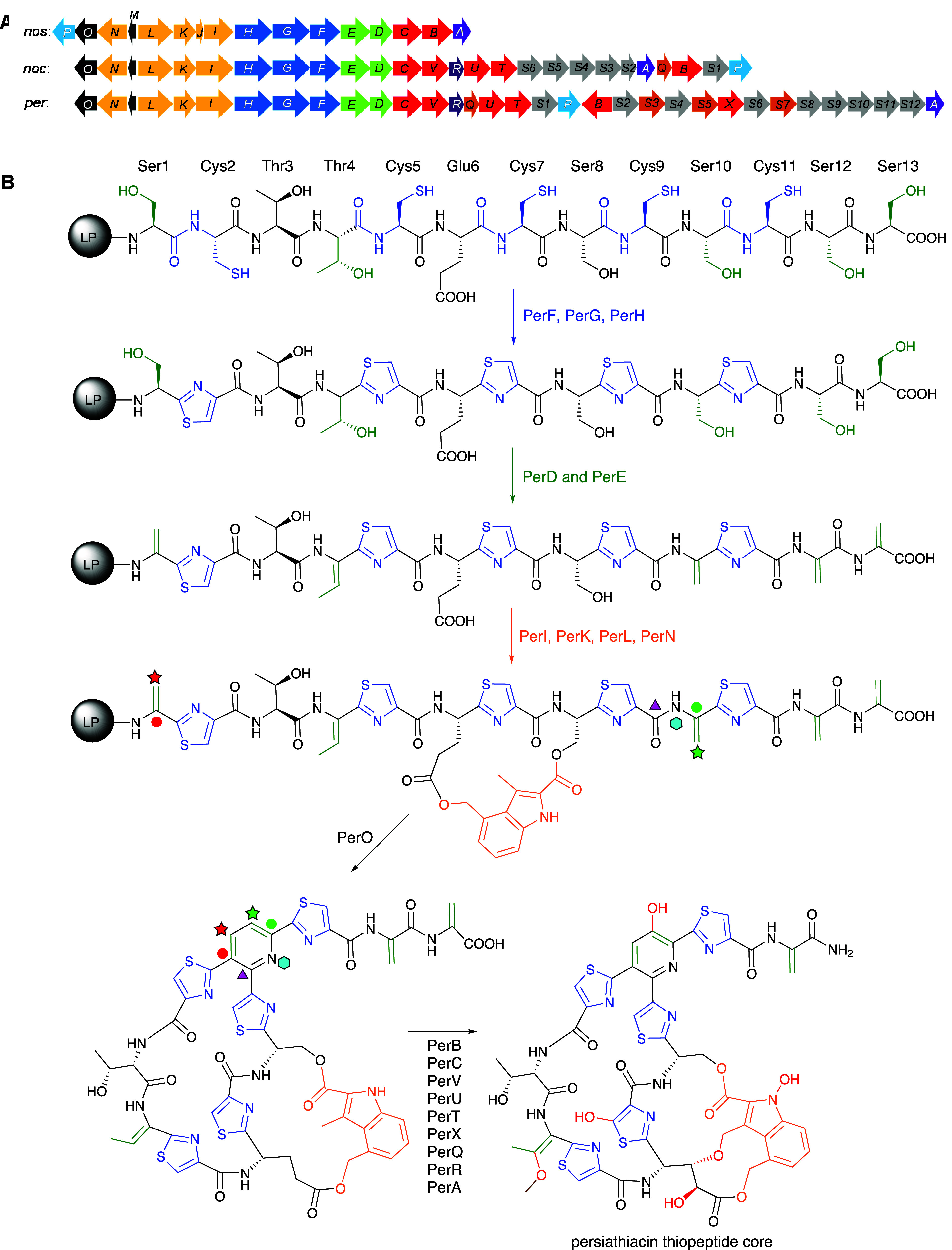
(A) Comparison of the nosiheptide (*nos*), nocathiacin (*noc*), and putative persiathiacin (*per*) biosynthetic gene clusters. Genes are colored as follows. Blue: thiazole formation; green: Ser/Thr dehydration; orange: DMIA formation and attachment; red: cytochromes P450; gray: 6-deoxysugar biosynthesis and attachment; brown: methyltransferases. (B) The biosynthesis of the thiopeptide core of the persiathiacins is proposed to commence with transcription and translation of *perM* to yield a precursor peptide comprised of an N-terminal leader peptide (LP) fused to a C-terminal core peptide (structure depicted). The core peptide undergoes a series of post-translational modifications catalyzed by several enzymes encoded by the persiathiacin biosynthetic gene cluster. See main text for further details.

To identify the persiathiacin BGC, the genome of *Actinokineospora* sp. UTMC 2448 was sequenced using single-molecule real-time (SMRT) sequencing. A complete circular genome sequence consisting of 7,012,397 bp was obtained using this approach (GenBank accession number CP031087). Analysis of the sequence using antiSMASH identified 32 putative specialized metabolite BGCs (Table S3).^25^ Among these, a cluster containing 33 genes (cluster 11; Table S3), several of which encode homologues of enzymes involved in the biosynthesis of other thiopeptides, was postulated to direct persiathiacin biosynthesis (Figure 3). Sequence comparisons showed that the products of *perA*–*perP* have a significant degree of similarity to the proteins encoded by *nosA*–*nosP* and *nocA*–*nocP* in the nosiheptide and nocathiacin BGCs, respectively (Table S4). Homologues of five additional genes in the nocathiacin BGC (*nocR* and *nocT–nocV*), absent from the nosiheptide cluster, are present in the putative persiathiacin cluster (*perR* and *perT–perV*, respectively; Figure 3). Moreover, the putative persiathiacin BGC contains 12 genes (*perS1–perS12*) hypothesized to be responsible for the biosynthesis and attachment of four 6-deoxysugars to the thiopeptide core (Table S4).

**Figure 4:**
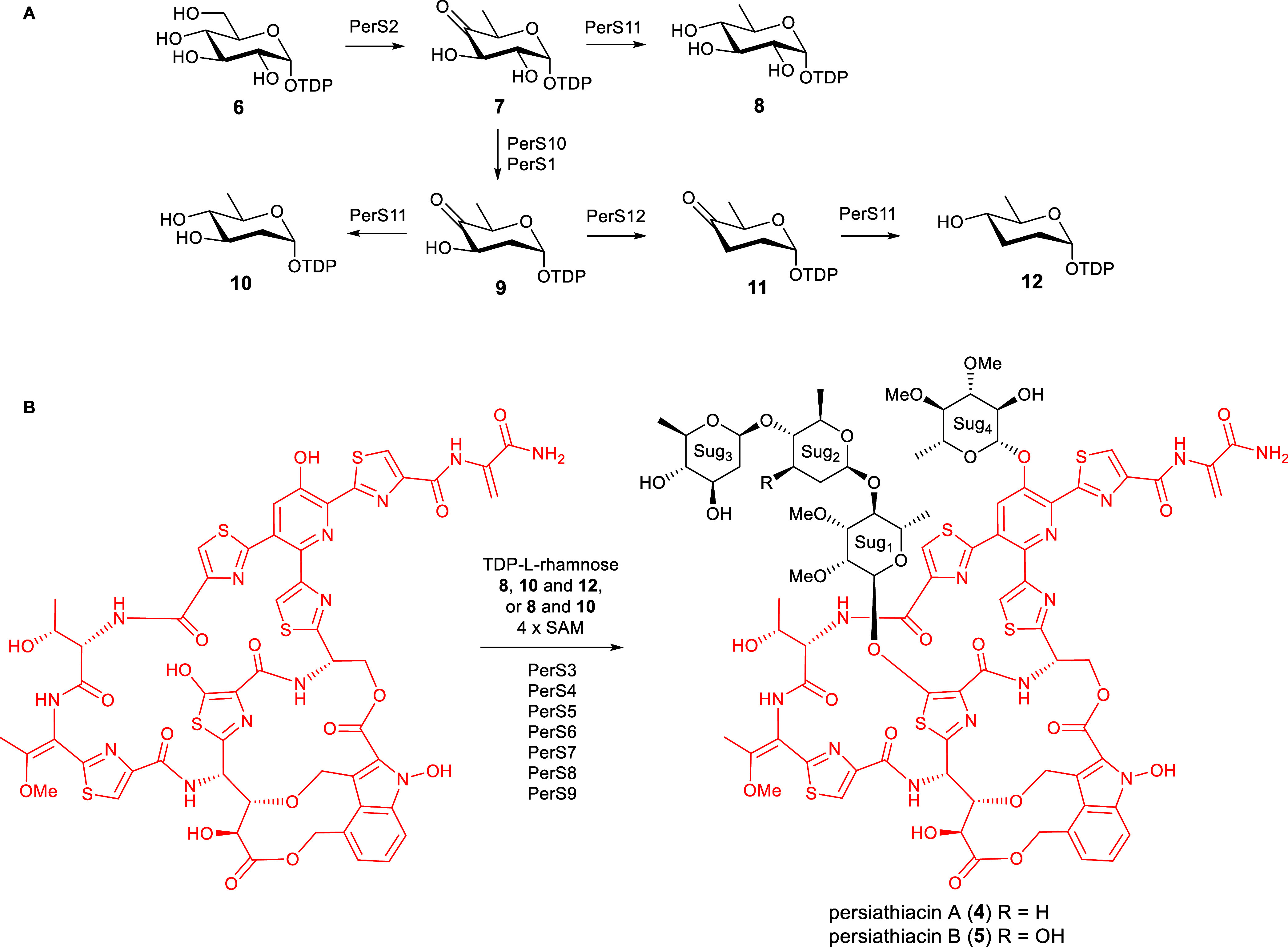
The enzymes encoded by *perS1*–*perS12* are proposed to be responsible for the biosynthesis and attachment of the 6-deoxysugars to the persiathiacin aglycone. (A) Proposed pathway for assembly of TDP-6-deoxy-α-d-glucose **8**, TDP-α-d-olivose **10**, and TDP-α-d-amicetose **12**. (B) The glycosyltransferases encoded by *perS4*, *perS6*, *persS8*, and *perS9* are proposed to decorate the persiathiacin core peptide with l-rhamnose, 6-deoxy-d-glucose, d-olivose, and d-amicetose (persiathiacin A **4**), or l-rhamnose, 6-deoxy-d-glucose, and d-olivose (persiathiacin B **5**). The methyltransferases encoded by *perS3*, *perS5*, and *perS7* are hypothesized to *O*-methylate the l-rhamnose and 6-deoxy-d-glucose residues to produce the mature antibiotics. The timing of these transformations remains to be determined.

Detailed sequence analysis of *perA–perV* and *perS1–perS12* enabled us to propose a biosynthetic pathway for persiathiacins A and B (Figures 3 and 4). First, *perM* is transcribed and translated into a 49 amino acid (aa) precursor peptide, consisting of a 36 aa N-terminal LP fused to a 13 aa C-terminal core peptide with the sequence SCTTCECSCSCSS, which is fully consistent with the thiopeptide core structure of the persiathiacins deduced from the spectroscopic data.

Cyclodehydration of the cysteine residues is proposed to be catalyzed by PerG and PerH, followed by dehydrogenation catalyzed by PerF to yield the five thiazoles in the persiathiacins.^7^ Putative dehydratases PerD and PerE are proposed to further modify the core peptide by catalyzing selective dehydration of Ser1, Ser10, Ser12, Ser13, and Thr4.

Four enzymes encoded by *perI*, *perK*, *perL*, and *perN* are proposed to be responsible for the production of 3,4-dimethylindolic acid (DMIA) from l-tryptophan and its attachment to the core peptide (Figures 3 and S15). PerL is a putative radical *S*-adenosylmethionine (SAM) enzyme that is homologous to NosL, which transforms l-tryptophan into 3-methyl-2-indolic acid (MIA) via unusual constitutional isomerization of a C–C bond.^26−34^ In nosiheptide biosynthesis, the ATP-dependent NosI enzyme adenylates MIA and loads it onto the phosphopantetheine thiol of the acyl carrier protein (ACP) NosJ. NosK then transfers the MIA residue to Cys8 of the nosiheptide core peptide (Figure S15).^35−37^ The persiathiacin and nocathiacin BGCs both lack *nosJ* homologues. Sequence comparisons of NosJ with PerI and PerK revealed similarity between NosJ and the C-terminus of PerK. Similarly, it has previously been noted that the C-terminus of NocK is homologous to NosJ.^35^ Thus, it appears that in persiathiacin and nocathiacin biosynthesis, the C-terminal ACP domains of PerK and NocK are loaded with MIA by PerI and NocI, respectively. The N-terminal domains of PerK and NocK then catalyze attachment of the MIA residue to Ser8 of the persiathiacin and nocathiacin core peptides, respectively (Figure S15). Subsequently, the putative radical SAM methylase PerN is proposed, by analogy with the well-characterized mechanism of NosN,^35^ to catalyze methylenation of C4 in the MIA residue. The resulting electrophilic intermediate is attacked by the Glu6 carboxylate to form an ester linkage.^38^ Finally, PerO, which has >50% sequence identity to NosO and NocO, is hypothesized to be responsible for formation of the macrocycle and pyridine in the persiathiacins via a [4 + 2] cycloaddition.^39,40^

**Figure 5:**
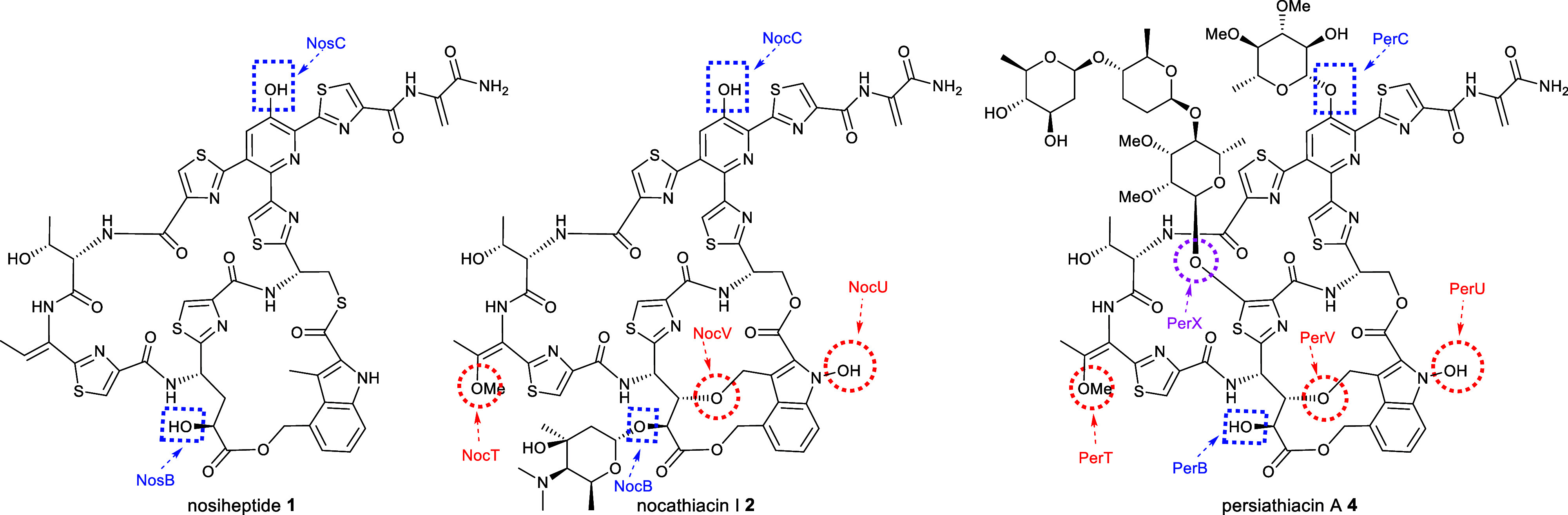
Comparative analysis of the structures of nosiheptide **1**, nocathiacin I **2**, and persiathiacin A **4** and the functions of the CYPs encoded by their BGCs. Blue dashed boxes highlight hydroxyl groups proposed to be installed by homologous CYPs (NosB/NocB/PerB and NosC/NocC/PerC) encoded by all three BGCs. Red dashed circles highlight hydroxyl groups proposed to be introduced by CYPs (NocT/PerT, NocV/PerV, and NocU/PerU) encoded by the nocathiacin and persiathiacin BGCs, but not the nosiheptide BGC. A purple dashed circle highlights the hydroxyl group proposed to be introduced by the CYP encoded by *perX*, which is only present in the persiathiacin BGC.

Of the six putative cytochromes P450 (CYPs) encoded by the persiathiacin BGC, two (PerB and PerC) are homologous to NosB/NocB and NosC/NocC, which hydroxylate C3 of Glu6 and the pyridine, respectively.^13^ The CYPs encoded by *perV*, *perU*, and *perT* are similar in sequence to NocV, NocU, and NocT, respectively, encoded by the nocathiacin BGC. Genes encoding homologues of these enzymes are absent from the nosiheptide BGC. PerV is proposed to perform an analogous function to NocV—i.e., formation of the ether linkage between the indole and core peptide via a mechanism yet to be elucidated.^41^ Similarly, PerU is hypothesized to catalyze *N*-hydroxylation of the indole, by analogy with the proposed function of NocU.^42^ Comparison of the structures of persiathiacin A, nocathiacin I and nosiheptide (Figure 4) suggests the putative CYPs encoded by *perT*/*nocT* and methyltransferases encoded by *perQ*/*nocQ* catalyze hydroxylation and subsequent *O*-methylation of the dehydrobutyrine residue to form the corresponding *O*-methyl-Dht residue. The only CYP-encoding gene in the persiathiacin BGC that does not have a homologue in either the nosiheptide or nocathiacin BGCs is *perX*. Structural comparison of persiathiacin A with nocathiacin I and nosiheptide suggests that the enzyme encoded by this gene catalyzes hydroxylation of C5 in thiazole 3, to create the attachment site for the trisaccharide (Figure 5).

The final enzyme proposed to be involved in the assembly of the thiopeptide core of the persiathiacins is PerA. This enzyme is homologous to NosA, which catalyzes dealkylative cleavage of the C-terminal Dha residue in nosiheptide biosynthesis, resulting in formation of the corresponding amide.^43^ PerA is proposed to catalyze an analogous reaction in persiathiacin biosynthesis (Figure 3).

The enzymes encoded by *perS1*–*perS12* are proposed to assemble the glycosyl residues and catalyze their attachment to the thiopeptide core of the persiathiacins. The biosynthesis of these 6-deoxysugars is proposed to commence with the conversion of thymine diphosphate (TDP)-α-d-glucose **6** to TDP-4-keto-6-deoxy-α-d-glucose **7** catalyzed by PerS2, which shows sequence similarity to TDP-glucose-4,6-dehydratases (Figure 4). At this point, the pathway appears to bifurcate. While PerS11, which is similar to TDP-hexose-4-ketoreductases, is proposed to catalyze the formation of TDP-6-deoxy-α-d-glucose **8** from TDP-4-keto-6-deoxy-α-d-glucose **7**, PerS10 and PerS1, which are homologues of TDP-4-keto-6-deoxy-d-glucose-2,3-dehydratases and TDP-4-keto-6-deoxy-d-glucose-3-ketoreductases, respectively, are hypothesized to convert **7** to TDP-4-keto-2,6-deoxy-α-d-glucose **9**. A further bifurcation then occurs. PerS11 catalyzes the conversion of TDP-4-keto-2,6-deoxy-α-d-glucose **9** to TDP-d-olivose **10**, whereas PerS12, which is similar to TDP-4-keto-2,6-deoxy-d-glucose-3-dehydratases, converts **9** to TDP-4-keto-2,3,6-deoxy-α-d-glucose **11**. Finally, PerS11 catalyzes the reduction of TDP-4-keto-2,3,6-deoxy-α-d-glucose **11** to TDP-d-amicetose **12**. This analysis is consistent with the assignment of sugars 2, 3, and 4 as d-amicetose, d-olivose, and 3,4-di-*O*-methyl-6-deoxy-β-d-glucose, respectively, in persiathicin A **4**, and the substitution of d-amicetose by a second d-olivose residue in persiathiacin B **5**.

Philipimycin **3**, which bears the highest degree of structural similarity among known thiopeptide antibiotics to the persiathiacins, is proposed to be decorated with d-amecitose and two *O*-methylated l-rhamnose derivatives.^17^ Moreover, no 6-deoxysugar biosynthetic genes, beyond those hypothesized to be involved in the assembly of TDP-d-amicetose, TDP-d-olivose, and TDP-6-deoxy-β-d-glucose, are present in the persiathiacin BGC (Figure 3 and Table S4). Because l-rhamnose is ubiquitously incorporated into bacterial cell surface carbohydrates,^44^ dedicated genes for TDP-l-rhamnose biosynthesis are invariably absent from rhamnosylated natural product BGCs. Taken together, these observations are consistent with the assignment of sugar 1 as an *O*-dimethylated l-rhamnose derivative. The deoxysugar residues contain a total of four methoxy groups (appended to C2 and C3 in sugar 1 and C3 and C4 in sugar 4), but only three putative *O*-methyltransferase-encoding genes (*perS3*, *perS5*, and *perS7*) are present in the persiathiacin BGC. It therefore appears that one of PerS3, PerS5, and PerS7 catalyzes the *O*-methylation of two distinct hydroxyl groups in the biosynthesis of one of these sugars.

Four genes (*perS4*, *perS6*, *perS8*, and *perS9*) encode putative glycosyltransferases, each of which is hypothesized to append one glycosyl residue to the thiopeptide core. Glycosyltransferases are known to possess broad substrate tolerance,^45^ explaining why small amounts of persiathiacin B **5**, in which sugar 2 is d-olivose rather than d-amicetose, are produced in addition to persiathiacin A **4**.

Given that (i) the core peptide sequence encoded by *perM* is in complete accord with the amino acid residues found in the thiopeptide core of persiathiacins A and B; (ii) the nocathiacins and persiathiacins have identical thiopeptide core structures, and the putative persiathiacin BGC encodes homologues of the full complement of enzymes needed to assemble the nocathiacin thiopeptide core from the precursor peptide; (iii) the persiathiacin BGC encodes an additional CYP (PerX) not found in the nocathiacin BGC, all the expected enzymes for assembly of the 6-deoxysugars in the persiathiacins, and four glycosyltransferases (compared to only one in the nocathiacin BGC), fully consistent with the tetraglycosylated structure of the persiathiacins; and (iv) the complete genome sequence of *Actinokineospora* sp. UTMC 2448 does not contain any other gene clusters with a significant similarity to known thiopeptide BGCs, it is highly probable that the BGC we have identified directs persiathiacin biosynthesis. Notwithstanding this, we endeavored to experimentally verify this hypothesis.

### PerX Catalyzes Hydroxylation of Nosiheptide

**Figure 6:**
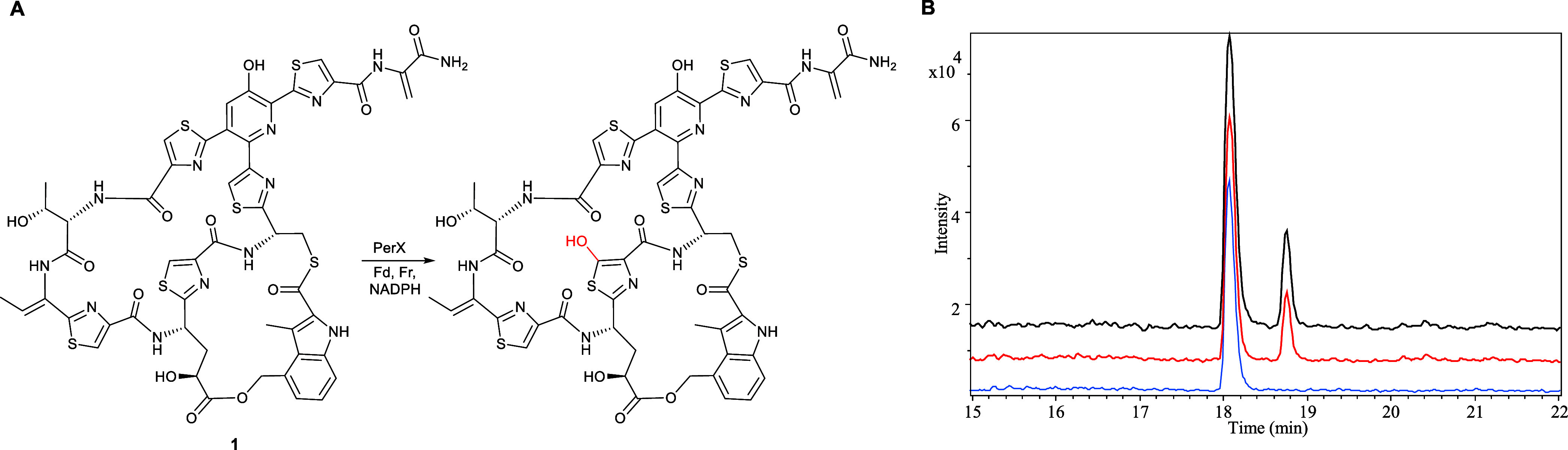
(A) Reaction catalyzed by purified recombinant PerX with nosiheptide **1**, in the presence of spinach ferredoxin (Fd), spinach ferredoxin reductase (Fr) and NADPH. The proposed site of oxygen atom insertion, based on the assigned function of PerX in persiathiacin biosynthesis, is highlighted in red. (B) Extracted ion chromatograms at *m*/*z* = 1222.1551 and 1238.1500, corresponding to [M + H]^+^ for nosiheptide and its hydroxylated derivative, respectively, from UHPLC–ESI-Q-TOF-MS analyses of: culture extracts of *S. actuosus* ATCC25421 expressing *perX* under the control of the strong constitutive ermE* promoter (top chromatogram); nosiheptide **1** after incubation for 3 h with purified recombinant PerX, Fd, Fr, and NADPH (middle chromatogram); and nosiheptide **1** after incubation for 3 h with heat-denatured PerX, Fd, Fr and NADPH (bottom chromatogram).

Due to a lack of genetic tools for the *Actinokineospora* genus, we were unable to obtain experimental evidence for the involvement of the nocathiacin-like BGC in *Actinokineospora* sp. UTMC 2448 in persiathiacin assembly via targeted disruption of one of the putative biosynthetic genes. Instead, we decided to investigate the ability of the putative CYP PerX to hydroxylate thiopeptides. Recombinant His_6_-tagged PerX was overproduced in *Escherichia coli* and purified using nickel-affinity chromatography. The identity of the purified protein, including the presence of a haem prosthetic group, was confirmed by ESI-Q-TOF-MS analysis (Figure S16). The purified protein was incubated with commercially available nosiheptide **1**, spinach ferredoxin, spinach ferredoxin reductase, and NADPH at room temperature for 3 h. UHPLC-ESI-Q-TOF-MS analysis of the reaction mixture revealed a species with *m*/*z* = 1238.1493, corresponding to the [M + H]^+^ ion for a compound with the molecular formula C_51_H_43_N_13_O_13_S_6_ (calculated *m*/*z* = 1238.1500 for C_51_H_44_N_13_O_13_S_6_^+^) that was absent from a control reaction containing heat-inactivated enzyme. The molecular formula of this species is consistent with the insertion of an oxygen atom into the nosiheptide backbone (measured *m*/*z* = 1222.1542; calculated *m*/*z* = 1222.1551 for C_51_H_44_N_13_O_12_S_6_^+^) (Figure 6). These data indicate that PerX can hydroxylate substrate analogues with significant modifications to the persiathiacin/nocathiacin core thiopeptide structure, suggesting it may hold promise for development into a new tool for targeted thiopeptide structural modification.

In an attempt to obtain sufficient quantities of the oxygenated nosiheptide derivative for NMR spectroscopic analysis, *perX* was expressed under the control of the constitutive ermE* promoter in the nosiheptide producer *Streptomyces actuosus* ATCC25421. Although the same nosiheptide derivative as that produced in the in vitro experiments was observed in UHPLC–ESI-Q-TOF-MS analyses of extracts from this strain (Figure 6), it was not possible to isolate sufficient quantities of the compound for full characterization by NMR spectroscopy.

### PerS4 Catalyzes Glycosylation of Nosiheptide

**Figure 7:**
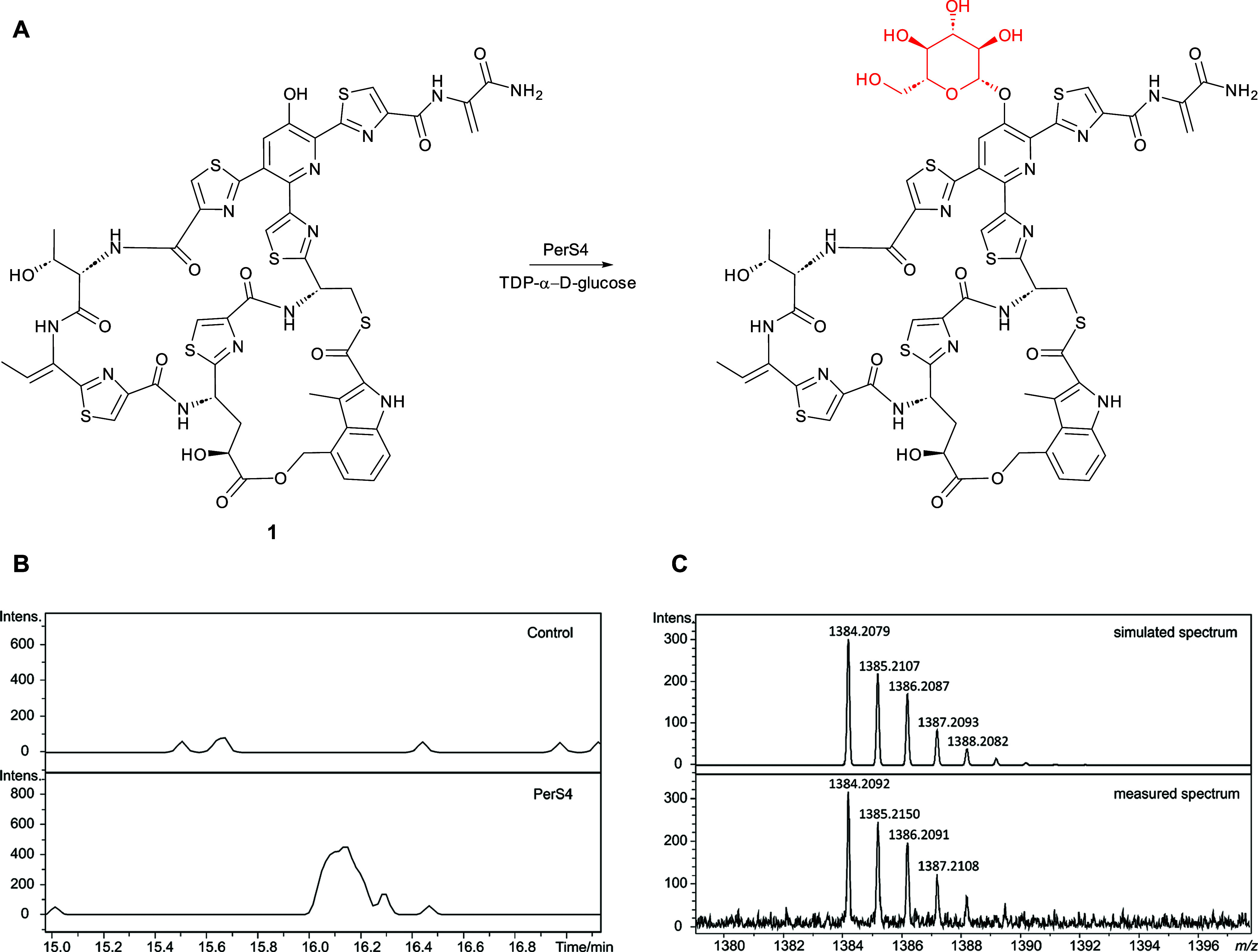
(A) Reaction proposed to be catalyzed by purified recombinant PerS4 with nosiheptide **1** and TDP-α-d-glucose. (B) Extracted ion chromatograms at *m*/*z* = 1384.2080, corresponding to [M + H]^+^ for glycosylated nosiheptide, from UHPLC–ESI-Q-TOF-MS analyses of incubations of nosiheptide **1** with TDP-α-d-glucose and PerS4 (bottom) and a control reaction containing heat-inactivated PerS4. (C) Comparison of the simulated mass spectrum for C_57_H_53_N_13_O_17_S_6_^+^ (top) with the measured spectrum of the species eluting at 16.1 min, corresponding to glycosylated nosiheptide (bottom).

To further validate the involvement of the identified gene cluster in persiathiacin biosynthesis, we investigated the ability of the putative glycosyltransferase PerS4 to glycosylate thiopeptides. A homologue of PerS4 from *Actinobacteria fastidiosa* JCM3276 has recently been reported to rhamnosylate nosiheptide.^46^ Recombinant His_6_-tagged PerS4 was overproduced in *E. coli* and purified using nickel-affinity chromatography and its identity was confirmed by ESI-Q-TOF-MS analysis (Figure S17). The purified protein was incubated with commercially available nosiheptide **1** and TDP-α-d-glucose at 30 °C for 12 h. UHPLC–ESI-Q-TOF-MS analysis of the reaction mixture revealed a species with *m*/*z* = 1384.2092, corresponding to the [M + H]^+^ ion for a compound with the molecular formula C_57_H_52_N_13_O_17_S_6_ (calculated *m*/*z* = 1384.2079 for C_57_H_53_N_13_O_17_S_6_^+^) that was absent from a control reaction containing heat-inactivated enzyme. The molecular formula of this species is consistent with the attachment of a d-glucose residue to one of the hydroxyl groups in nosiheptide (Figure 7). Nosiheptide **1** contains the 3-hydroxypyridine moiety that is glycosylated with the dimethylated 6-deoxy-d-glucose derivative in the persiathiacins but lacks the hydroxylated thiazole that serves as the attachment site for the trisaccharide. We therefore tentatively conclude that PerS4 catalyzes transfer of the d-glucose residue to the hydroxypyridine moiety of nosiheptide (Figure 7), but further experiments will be required to confirm this.

### Biological Activity

Persiathiacin A was tested against the ESKAPE panel of pathogens by measuring minimum inhibitory concentrations (MICs).^47^ Persiathiacin A showed potent activity against MRSA (MIC of 0.025 μg/mL) and moderate activity against *Enterococcus faecium* (MIC of 32 μg/mL). The compound was inactive against all Gram-negative bacteria in the panel up to clinically relevant MIC cutoffs (Table 1). As nocathiacin has been reported to be active against drug-susceptible and resistant clinical strains of *M. tuberculosis*,^48,49^ we evaluated the activity of persiathiacin A against several clinical isolates of *M. tuberculosis* using the resazurin microtiter assay.^50^ It was found to be active against all isolates tested, including the drug-susceptible strain H37Rv, four isoniazid-resistant strains, and strains CHUV80059744 and CHUV80037024 resistant to both isoniazid and rifampicin (Table 2). Persiathiacin A exhibited negligible toxicity toward the A2780 ovarian cancer cell line up to the maximum tested concentration of 400 μM (Figure S18).

**Table 1:** MIC Values (μg/mL) of Persiathiacin A Against ESKAPE Pathogens

ESKAPE pathogen	persiathiacin A
*E. faecium*	32
*S. aureus*	0.025
*Klebsiella pneumoniae*	>64
*Acinetobacter baumannii*	>64
*Pseudomonas aeruginosa*	>64
*Enterobacter* sp.	>64

**Table 2:** MIC Values (μg/mL) of Persiathiacin A, Rifampicin and Isoniazid Against *M. tuberculosis* Isolates

*M. tuberculosis*	mutation	resistance	persiathiacin A	rifampicin	isoniazid
H37Rv	none	none	3.6	0.0008	0.04
HUGMB6726	*inhA*	isoniazid	3.7	0.0008	2.2
HUGMB3649	*inhA*	isoniazid	3.9	0.001	0.08
CHUV80045776	*katG*	isoniazid	1.6	0.001	2.5
HUGMI1020	*katG*	isoniazid	1.7	0.002	1.3
CHUV80059744	*rpoB* and *katG*	isoniazid, rifampicin	2.2	14	>10
CHUV80037024	*rpoB*, *katG*, and *inhA*	isoniazid, rifampicin	3.1	>10	>10

The antibacterial activity of several thiopeptides is known to result from inhibition of ribosomal protein biosynthesis.^51−53^ In the accompanying manuscript,^54^ we report an investigation of the mechanisms of action and resistance to persiathiacin A, revealing that, in common with other 26-membered macrocycle-containing thiopeptides, it inhibits translation elongation by targeting ribosomal protein L11.

## Conclusions

Despite their potent antibacterial activity, the development of thiopeptides into clinically useful antibiotics has been prevented by their poor pharmacological properties, particularly their low aqueous solubility. Glycosylation is a widely used strategy for increasing the solubility of therapeutic peptides. However, most naturally occurring thiopeptides are either unglycosylated, or have a single sugar attached to the γ-hydroxyl group of the modified Glu residue, limiting opportunities to create novel glycosylated derivatives of thiopeptides via biosynthetic engineering. The sole exception, prior to this work, was philipimycin A, which has a trisaccharide appended to the central thiazole. Although philipimycin A is a rare example of thiopeptide that is active in vivo,^10^ nothing is known about its biosynthesis.

The discovery and biosynthetic elucidation of thiopeptides with novel glycosylation patterns could provide a useful foundation for the creation of new polyglycosylated thiopeptide derivatives with greater aqueous solubility and enhanced therapeutic potential. Our discovery in this work of the polyglycosylated thiopeptides persiathiacins A and B from *Actinokineospora* sp. UTMC 2448, and the gene cluster responsible for their biosynthesis, is therefore significant for several reasons. First, the persiathiacins are the first examples of naturally occurring thiopeptides with a sugar appended to the hydroxpyridine. Glycosylation of the hydroxypyridine in nocathician has been reported to significantly improve aqueous solubility.^11^ The identification of the persiathiacin BGC opens the path for the development of biosynthetic engineering approaches to the creation of novel thiopeptide derivatives bearing glycosylated hydroxypyridines, as indicated by our demonstration that PerS4 is able to append a glucose residue to nosiheptide. Second, the identification of the persiathiacin BGC reveals the molecular mechanism for attachment of a trisaccharide to the central thiazole of thiopeptides. The incorporation of different sugars into the trisaccharides appended to the central thiazoles of philipimycin A, persiathiacin A, and persiathiacin B suggests the glycosylation machinery is substrate tolerant. Thus, biosynthetic engineering could be used to create a range of thiopeptide analogues with various mono-, di- and trisaccharides attached to the central thiazole. Third, the observation that nocathiacin I, philipimycin A, and persiathiacin A all display strong activity against *S. aureus* and *M. tuberculosis*, despite their diverse glycosylation patterns, indicates that creation and biological evaluation of novel glycosylated thiopeptide derivatives may be a fruitful strategy for circumventing the historical problems that have prevented this class of antibiotics from progressing into clinical application.

## Materials and Methods

### General Experimental Procedures

Optical rotations were measured on an Optical Activity Ltd. AA-1000 millidegree autoranging polarimeter (589 nm). Specific rotations are given in units of 10^–1^ deg cm^2^ g^–1^. UV spectra were acquired on a PerkinElmer Lambda 35 UV/vis spectrophotometer. IR spectra were recorded on an Alpha Bruker Platinum ATR single reflection diamond ATR module. UHPLC-ESI-Q-TOF-MS analyses were performed using a Dionex UltiMate 3000 UHPLC connected to a Zorbax Eclipse Plus C18 column (100 × 2.1 mm, 1.8 μm) coupled to a Bruker MaXis IMPACT, or MaXis II mass spectrometers. Mobile phases consisted of water (A) and acetonitrile (B), each supplemented with 0.1% formic acid. A gradient of 5–100% B over 30 min was employed at a flow rate of 0.2 mL/min. The mass spectrometer was operated in positive ion mode with a scan range of 50–3000 *m*/*z*. Calibration was performed with 1 mM sodium formate through a loop injection of 20 μL at the start of each run. Persiathiacins A and B were dissolved in a mixture of CDCl_3_–CD_3_OD (9:1) for NMR spectroscopic analyses. NMR spectra were recorded on Bruker 500 or 700 MHz spectrometers equipped with DCH and TCl cryoprobes, respectively, at 25 °C. The ^1^H and ^13^C NMR chemical shifts were referenced to the solvent peaks at δ_H_ 7.26 and δ_C_ 77.16 for CDCl_3_. All HPLC and LC–MS experiments were performed with the MeCN–H_2_O gradient solvent system. Millipore Milli-Q H_2_O and HPLC grade solvents were used for chromatography.

### Strain Isolation and Identification

*Actinokineospora* sp. UTMC 2448 was isolated from mud sample collected from Bushehr, Iran. The sample was dried at 50 °C, ground to a powder and passed through a 2 mm sieve. Strains were isolated on solid Reasoner’s 2A medium^55^ after 3 weeks of incubation at 28 °C. Solid ISP2 medium was then used to purify the strains. Purified strains were preserved in 30% glycerol at −70 °C. To identify the strains, 16S rRNA genes were amplified using a set of universal primers (27F, 1100F, 1100R, 1525R). Amplified DNA obtained from the reactions was purified using a PCR purification kit (Roti-Prep PCR Purification). The 16S rRNA gene sequence of the strains was BLASTed against the GenBank and EzTaxon databases.^56^

### Production, Extraction, and HPLC Purification of Persiathiacins A and B

*Actinokineospora* sp. UTMC 2448 was grown on solid ISP2 medium (4 g/L glucose, 4 g/L yeast extract, 10 g/L malt extract, 2 g/L CaCO_3_, 15 g/L Bacto agar) for 7 days at 30 °C. The agar cultures were chopped and extracted with EtOAc. The extract was dried on a rotary evaporator and preadsorbed to C18-bonded silica, and then packed into a stainless steel HPLC guard cartridge (10 × 30 mm) attached to a semipreparative reverse-phase C18 Betasil column (21.2 × 150 mm). The column was eluted with 5% acetonitrile for 5 min, then a linear gradient from 5 to 100% acetonitrile was applied over 45 min, and the column was eluted for an additional 10 min with 100% acetonitrile. The flow rate was 9 mL/min. Sixty fractions were collected in 1 min increments over 60 min. Pure persiathiacin A was obtained in fraction 35. Persiathiacin B was purified from a mixture of persiathiacins A and B in fraction 34 using a reverse-phase C18 Betasil column (21.2 × 150 mm). Isocratic elution with 40% acetonitrile for 5 min followed by a linear gradient to 65% over 45 min was used to achieve separation of persiathiacin B (fraction 26) from persiathiacin A (fraction 28).

#### Persiathiacin A

Amorphous solid (19 mg); [α]_D_^26^ + 86 (*c* 0.05, CHCl_3_/MeOH (9:1)); UV (CHCl_3_/MeOH (9:1)) λ_max_ (log ε) 231 (5.85), 278 (5.65), 353 (5.25) nm; IR ν_max_ 3392, 2980, 1725, 1667, 1534, 1250, 1205, 1121, 1065, 751 cm^–1^; ^1^H NMR (500 MHz, CDCl_3_/CD_3_OD (9:1)) and ^13^C NMR (125 MHz, CDCl_3_/CD_3_OD (9:1)), see Table S1; HRESIMS *m*/*z* 1874.4638 [M + H]^+^ (calcd for C_80_H_92_N_13_O_30_S_5_, 1874.4671).

#### Persiathiacin B

Amorphous solid (1.8 mg); [α]_D_^26^ + 86 (*c* 0.05, CHCl_3_/MeOH (9:1)); UV (CHCl_3_/MeOH (9:1)) λ_max_ (log ε) 232 (5.86), 275 (5.61), 353 (5.21) nm; IR ν_max_ 3402, 2970, 1667, 1533, 1250, 1205, 1121, 1067, 1037, 751 cm^–1^; ^1^H NMR (700 MHz, CDCl_3_/CD_3_OD (9:1)) and ^13^C NMR (175 MHz, CDCl_3_/CD_3_OD (9:1)), see Table S2; HRESIMS *m*/*z* 1890.4611 [M + H]^+^ (calcd for C_80_H_92_N_13_O_31_S_5_, 1890.4620).

### PacBio Library Preparation and Sequencing

Genomic DNA was extracted from *Actinokineospora* sp. UTMC2448. A SMRTbell template library was prepared according to the manufacturer’s instructions (Pacific Biosciences, Menlo Park, CA, USA), following the Procedure & Checklist—Greater Than 10 kb Template Preparation. Briefly, for preparation of 15 kb libraries, 8 μg genomic DNA was sheared using g-tubes (Covaris, Woburn, MA, USA) according to the manufacturer's instructions. DNA was end-repaired and ligated overnight to hairpin adapters by applying components from the DNA/Polymerase Binding Kit P6 (Pacific Biosciences, Menlo Park, CA, USA). Reactions were carried out according to the instructions of the manufacturer. BluePippin size-selection to greater than 4 kb was performed according to the manufactureŕs instructions (Sage Science, Beverly, MA, USA). Conditions for annealing of sequencing primers and binding of polymerase to purified SMRTbell template were assessed with the Calculator in RS Remote (Pacific Biosciences, Menlo Park, CA, USA). SMRT sequencing was carried out on the PacBio *RSII* (Pacific Biosciences, Menlo Park, CA, USA), taking one 240 min movie for one SMRT cell using the P6 Chemistry. Sequencing resulted in 76,562 postfiltered reads with a mean read length of 10,180 bp.

### Genome Assembly, Error Correction, and Annotation

SMRT cell data were assembled using the “RS_HGAP_Assembly.3” protocol included in SMRT Portal version 2.3.0 using default parameters. The assembly resulted in a single circular chromosome. Error correction was performed by a mapping of 7 million paired-end Illumina reads of 2 × 100 bp onto the genome using BWA (PMID 19451168)^57^ with subsequent variant and consensus calling using VarScan (PMID 22300766).^58^ A consensus concordance of QV60 could be confirmed for the genome. Finally, annotation was carried out using Prokka 1.8 (PMID 24642063).^59^ Prediction of specialised metabolite BGCs was made using antiSMASH v3.0 (Table S3). The putative persiathiacin biosynthetic gene cluster was subjected to detailed manual annotation via comparative sequence analysis (Table S4).

### Overproduction and Purification of PerX

The gene encoding PerX was PCR-amplified from *Actinokineospora* sp*.* UTMC 2448 gDNA using Phusion DNA polymerase (NEB) and primers 5′-GTGCCGCGCGGCAGCCATATGCTTCCCGAGCCGTACACCCCCGAGTTCT-3′ and 5′-TCGACGGAGCTCGAATTCTCATCGCGTCACCCGCAGCTCGGCCA-3′ (regions complementary to the gene sequence underlined). The linear pET28a (NEB) vector backbone was PCR-amplified with primers 5′-TGAGAATTCGAGCTCCGTCGACAAGCTTG-3′ and 5′-CATATGGCTGCCGCGCGGCAC-3′. PCR products were separated on a 1% agarose gel and bands were excised and purified with a GeneJET Gel Extraction Kit (Thermo Scientific). Cloning of the pure insert into the *Nde*I/*Eco*RI restriction sites of the linear pET28a vector was accomplished by Gibson assembly following the manufacturer’s instructions (NEB). The resulting vector was used to transform *E. coli* TOP10 cells (Invitrogen) and plated on LB agar containing kanamycin (50 μg/mL). Colonies were picked and grown overnight in liquid LB medium. Plasmids were isolated from the culture using a GeneJET Plasmid Miniprep Kit (Thermo Scientific) and inserts were sequenced to verify their integrity. The correct pET28a plasmid containing *perX* was used to transform *E. coli* BL21(DE3) cells. A single colony was used to inoculate liquid LB medium (10 mL) containing kanamycin (50 μg/mL), which was incubated overnight at 37 °C and 180 rpm; this was then used to further inoculate liquid LB medium (1 L) containing kanamycin (50 μg/mL). The resulting culture was incubated at 37 °C and 180 rpm until OD_595 nm_ reached 0.6, then IPTG (0.5 mM) was added, and expression was continued overnight at 15 °C and 180 rpm. The cells were harvested by centrifugation (5000 rcf, 20 min, 4 °C) and resuspended in buffer (30 mM HEPES, 500 mM NaCl, 10% glycerol, pH 7.5) at 20 mL/L of growth medium, then lysed using sonication (Vibra-Cell Ultrasonic Liquid Processor; Sonics & Materials, Inc.). The lysate was centrifuged (30,000 rcf, 60 min, 4 °C) and the resulting supernatant was passed through a 0.45 μm filter (Sartorius). An ÄKTA pure FPLC (GE Healthcare) was used to purify PerX as follows. The supernatant was loaded onto a 1 mL HisTrap HP column (GE Healthcare), which had been equilibrated with resuspension buffer (30 mM HEPES, 500 mM NaCl, 10% glycerol, pH 7.5). Proteins were eluted in a stepwise manner using increasing concentrations of imidazole (0–150 mM) in resuspension buffer. The presence of the protein of interest in the elution fractions was confirmed by SDS–PAGE. Fractions containing the pure protein were pooled and concentrated to ∼100 μM using a 50 kDa MWCO Vivaspin centrifugal concentrator (Sartorius). Aliquots of 50 μL were snap-frozen in liquid N_2_ and stored at −80 °C until further use.

### Hydroxylation of Nosiheptide by PerX

A 200 μL reaction mixture containing nosiheptide (100 μM), spinach ferredoxin-NADP^+^ reductase (0.1 U/mL), spinach ferredoxin (50 μg/mL), NADPH (1 mM), and PerX (10 μM) in Tris–HCl (25 mM, pH 8) was incubated at room temperature for 3 h. The reaction was terminated by adding 200 μL of methanol, and after separating the precipitate by centrifugation (16,000 rcf, 10 min) the supernatant was analyzed by UHPLC–ESI-Q-TOF-HRMS. For the negative control, PerX was inactivated by boiling at 100 °C for 15 min.

### Expression of *perX* in the Nosiheptide-Producing Strain *S. actuosus*

*perX* was amplified from *Actinokineospora* sp*.* UTMC 2448 gDNA using Phusion DNA polymerase (NEB) and primers 5′-CAGCATATGGTGCTTCCCGAGCCGTAC-3′ and 5′-GACGAATTCTCATCGCGTCACCCGC-3′. The PCR product was digested with *Nde*I and *Eco*RI and cloned into the corresponding sites of pIB139 under the control of the ermE* constitutive promoter. The integrity of the construct was confirmed by sequencing and the resulting plasmid was used to transform *E. coli* ET12567/pUZ8002 cells by electroporation. A mixture of apramycin (50 μg/mL), kanamycin (50 μg/mL) and chloramphenicol (35 μg/mL) was used for selection on LB agar. The pIB139 vector containing *perX* was then introduced by conjugation into *S. actuosus* ATCC25421. The overnight culture was plated on SFM agar medium and overlaid with 1 mL of antibiotic solution mixture containing apramycin (50 μg/mL) and nalidixic acid (25 μg/mL). After 3 days, four colonies were picked and spread separately onto SFM agar medium containing apramycin (50 μg/mL) and nalidixic acid (25 μg/mL) and then further subcultured on five plates to produce spores. Spores from the resulting stocks were cultured in liquid medium containing corn steep liquor (10 g/L), soy flour (20 g/L), yeast extract (3 g/L), NaCl (4 g/L), KNO_3_ (0.2 g/L), CaCO_3_ (4 g/L), pH 7.0. Production of the hydroxylated nosiheptide derivative was confirmed by UHPLC–ESI-Q-TOF-MS analysis.

### Overproduction, Purification and Characterization of PerS4

The gene encoding PerS4 was synthesized and cloned into pET28a (+) by GenScript. The resulting C-terminal hexa-histidine fusion protein was overproduced in *E. coli* BL21(DE3) as described for PerX, except 0.4 mM IPTG was used and the culture was incubated at 15 °C for 16 h. The cells were lysed and the protein was purified from the cell lysate as described for PerX, except the cell lysate was suspended in 20 mM Tris–HCl, 100 mM NaCl, pH 8.0 and the protein was eluted stepwise using increasing concentrations of imidazole buffer (20–300 mM).

200 μM of purified recombinant PerS4 was incubated with 150 μL of nosiheptide in DMSO (2.4 mg/mL), 150 μL of a solution of TDP-α-d-glucose, prepared from thymidine monophosphate (27 mM) and α-d-glucose-1-phosphate as described previously,^50^ in 50 mM Tris–HCl (pH 7.5, total volume 1 mL). After incubation at 30 °C for 12 h the reaction was quenched by the addition of an equal volume of methanol. The precipitate was removed by centrifugation at 12,000 rpm for 10 min, and the supernatant was analyzed by UHPLC–ESI-Q-TOF-HRMS.

### MIC Assays Against *M. tuberculosis*

DMSO, glycerol, isoniazid, resazurin sodium salt, and rifampicin were purchased from Sigma-Aldrich (USA). Middlebrook 7H9 was purchased from Difco (USA) and albumin dextrose catalase from Chemie Brunschwig AG (Switzerland). The *M. tuberculosis* reference strain H37Rv was obtained from Institut Pasteur, Paris, and clinical specimens from patients were obtained from the Lausanne University Hospital (CHUV) and Geneva University Hospital (HUG).

The resazurin reduction microplate assay was performed as described previously.^50^ 2-fold serial dilutions of each test compound were prepared in 96-well plates from 10 mg/mL stocks in DMSO. Frozen aliquots of replicating tubercule bacilli (reference strains and clinical isolates) were thawed and diluted to an OD_600_ of 0.0001 (3×10^4^ cells/mL) and added to the plates to obtain a total volume of 100 μL. Plates were incubated for 6 days at 37 °C before adding resazurin (0.025% w/v to 1/10 of well volume). After overnight incubation, fluorescence of the resazurin metabolite resorufin was determined by excitation at 560 nm and emission at 590 nm, as measured by a TECAN infinite M200 microplate reader. The MIC was defined visually as the lowest concentration to prevent resazurin turnover from blue to pink and was confirmed by the level of measured fluorescence. MIC values were calculated using GraphPad Prism version 7.0 (GraphPad Software, Inc., La Jolla, CA, USA). The experiment was performed twice, and all the compounds were tested in triplicate (total of six replicates).

### Cytotoxicity Assays

Evaluation of the cytotoxicity of persiathiacin A was carried out using A2780 ovarian cancer cells, which were obtained from the European Collection of Cell Cultures. Cells were grown as adherent monolayers using Roswell Park Memorial Institute medium (RPMI 1640) supplemented with 10% v/v of fetal calf serum, 1% v/v of 2 mM glutamine and 1% v/v penicillin/streptomycin using a 5% CO_2_ humidified atmosphere. Cultures were regularly passaged when achieving 70–80% confluence. For these experiments, cells were seeded in a 96-well plate at a density of 5000 cells/well and allowed to attach for 48 h in persiathiacin-free medium. Various concentrations of persiathiacin were added in concentrations of up to 400 μM. Working solutions were obtained by dilution with cell culture medium from a 5% v/v DMSO/RPMI stock. After 24 h of drug exposure, cells were washed, and fresh medium was replenished to allow for 72 h of recovery time. Cell viability was assessed using the MTT assay. Formazan absorbance at 570 nm was recorded in a FLUOstar Omega microplate reader. In all cases, reported values were obtained as duplicates of triplicates in independent experiments with their associated standard deviations.

## Supporting Information Available

NMR data and spectra of persiathiacins A and B; proteins encoded by genes in the *per* BGC and their percentage identity to proteins of known function; comparative biosynthesis and attachment of MIA to the processed core peptides of nosiheptide, persiathiacin, and nocathiacin; Mass spectra of purified recombinant PerX and PerS4 (PDF)

## References

[ref1] Global Tuberculosis Report; World Health Organization: Geneva, 2021.

[ref2] Treatment of Tuberculosis: Guidelines, 4th ed.; World Health Organization: Geneva, 2010. 23741786

[ref3] Dashti Y.; Grkovic T.; Quinn R. J. Predicting natural product value, an exploration of anti-TB drug space. Nat. Prod. Rep. 2014, 31 (8), 990–998. 10.1039/C4NP00021H. 24881816

[ref4] Arnison P. G.; Bibb M. J.; Bierbaum G.; Bowers A. A.; Bugni T. S.; Bulaj G.; Camarero J. A.; Campopiano D. J.; Challis G. L.; Clardy J.; Cotter P. D.; Craik D. J.; Dawson M.; Dittmann E.; Donadio S.; Dorrestein P. C.; Entian K.-D.; Fischbach M. A.; Garavelli J. S.; Göransson U.; Gruber C. W.; Haft D. H.; Hemscheidt T. K.; Hertweck C.; Hill C.; Horswill A. R.; Jaspars M.; Kelly W. L.; Klinman J. P.; Kuipers O. P.; Link A. J.; Liu W.; Marahiel M. A.; Mitchell D. A.; Moll G. N.; Moore B. S.; Müller R.; Nair S. K.; Nes I. F.; Norris G. E.; Olivera B. M.; Onaka H.; Patchett M. L.; Piel J.; Reaney M. J. T.; Rebuffat S.; Ross R. P.; Sahl H.-G.; Schmidt E. W.; Selsted M. E.; Severinov K.; Shen B.; Sivonen K.; Smith L.; Stein T.; Süssmuth R. D.; Tagg J. R.; Tang G.-L.; Truman A. W.; Vederas J. C.; Walsh C. T.; Walton J. D.; Wenzel S. C.; Willey J. M.; van der Donk W. A. Ribosomally synthesized and post-translationally modified peptide natural products: overview and recommendations for a universal nomenclature. Nat. Prod. Rep. 2013, 30 (1), 108–160. 10.1039/C2NP20085F. 23165928 PMC3954855

[ref5] Ortega M. A.; van der Donk W. New Insights into the Biosynthetic Logic of Ribosomally Synthesized and Post-translationally Modified Peptide Natural Products. Cell Chem. Biol. 2016, 23 (1), 31–44. 10.1016/j.chembiol.2015.11.012. 26933734 PMC4779184

[ref6] Burkhart B. J.; Hudson G. A.; Dunbar K. L.; Mitchell D. A. A prevalent peptide-binding domain guides ribosomal natural product biosynthesis. Nat. Chem. Biol. 2015, 11 (8), 564–570. 10.1038/nchembio.1856. 26167873 PMC4509860

[ref7] Li C.; Kelly W. L. Recent advances in thiopeptide antibiotic biosynthesis. Nat. Prod. Rep. 2010, 27 (2), 153–164. 10.1039/B922434C. 20111801

[ref8] Zhang Q.; Liu W. Biosynthesis of thiopeptide antibiotics and their pathway engineering. Nat. Prod. Rep. 2013, 30 (2), 218–226. 10.1039/C2NP20107K. 23250571

[ref9] Hudson G. A.; Zhang Z.; Tietz J. I.; Mitchell D. A.; van der Donk W. A. In Vitro Biosynthesis of the Core Scaffold of the Thiopeptide Thiomuracin. J. Am. Chem. Soc. 2015, 137 (51), 16012–16015. 10.1021/jacs.5b10194. 26675417 PMC4819586

[ref10] Zhang Z.; Hudson G. A.; Mahanta N.; Tietz J. I.; van der Donk W. A.; Mitchell D. A. Biosynthetic Timing and Substrate Specificity for the Thiopeptide Thiomuracin. J. Am. Chem. Soc. 2016, 138 (48), 15511–15514. 10.1021/jacs.6b08987. 27700071 PMC5148741

[ref11] Wever W. J.; Bogart J. W.; Bowers A. A. Identification of Pyridine Synthase Recognition Sequences Allows a Modular Solid-Phase Route to Thiopeptide Variants. J. Am. Chem. Soc. 2016, 138 (41), 13461–13464. 10.1021/jacs.6b05389. 27575591

[ref12] Bewley K. D.; Bennallack P. R.; Burlingame M. A.; Robison R. A.; Griffitts J. S.; Miller S. M. Capture of micrococcin biosynthetic intermediates reveals C-terminal processing as an obligatory step for in vivo maturation. Proc. Natl. Acad. Sci. U.S.A. 2016, 113 (44), 12450–12455. 10.1073/pnas.1612161113. 27791142 PMC5098666

[ref13] Liu W.; Xue Y.; Ma M.; Wang S.; Liu N.; Chen Y. Multiple oxidative routes towards the maturation of nosiheptide. ChemBioChem 2013, 14 (13), 1544–1547. 10.1002/cbic.201300427. 23939763

[ref14] Bagley M. C.; Dale J. W.; Merritt E. A.; Xiong X. Thiopeptide Antibiotics. Chem. Rev. 2005, 105 (2), 685–714. 10.1021/cr0300441. 15700961

[ref15] Haste N. M.; Thienphrapa W.; Tran D. N.; Loesgen S.; Sun P.; Nam S.-J.; Jensen P. R.; Fenical W.; Sakoulas G.; Nizet V.; Hensler M. E. Activity of the thiopeptide antibiotic nosiheptide against contemporary strains of methicillin-resistant *Staphylococcus aureus*. J. Antibiot. 2012, 65 (12), 593–598. 10.1038/ja.2012.77. PMC3528839 23047246

[ref16] Pucci M. J.; Bronson J. J.; Barrett J. F.; DenBleyker K. L.; Discotto L. F.; Fung-Tomc J. C.; Ueda Y. Antimicrobial evaluation of nocathiacins, a thiazole peptide class of antibiotics. Antimicrob. Agents Chemother. 2004, 48 (10), 3697–3701. 10.1128/AAC.48.10.3697-3701.2004. 15388422 PMC521901

[ref17] Zhang C.; Occi J.; Masurekar P.; Barrett J. F.; Zink D. L.; Smith S.; Onishi R.; Ha S.; Salazar O.; Genilloud O.; Basilio A.; Vicente F.; Gill C.; Hickey E. J.; Dorso K.; Motyl M.; Singh S. B. Isolation, structure, and antibacterial activity of philipimycin, a thiazolyl peptide discovered from *Actinoplanes philippinensis* MA7347. J. Am. Chem. Soc. 2008, 130 (36), 12102–12110. 10.1021/ja803183u. 18698773

[ref18] Just-Baringo X.; Albericio F.; Álvarez M. Thiopeptide Engineering: A Multidisciplinary Effort towards Future Drugs. Angew. Chem., Int. Ed. 2014, 53 (26), 6602–6616. 10.1002/anie.201307288. 24861213

[ref19] Li W.; Leet J. E.; Lam K. S. Nocathiacin antibiotic derivatives prepared by microbial biotransformation. WO 014100, 2000.

[ref20] Li Y.; Clark K. A.; Tan Z. Methods for engineering therapeutic peptides. Chin. Chem. Lett. 2018, 29 (7), 1074–1078. 10.1016/j.cclet.2018.05.027.

[ref21] Chandrashekar C.; Hossain M. A.; Wade J. D. Chemical Glycosylation and Its Application to Glucose Homeostasis-Regulating Peptides. Front. Chem. 2021, 9 (222), 650025 10.3389/fchem.2021.650025. 33912539 PMC8072350

[ref22] Leet J. E.; Li W.; Ax H. A.; Matson J. A.; Huang S.; Huang R.; Cantone J. L.; Drexler D.; Dalterio R. A.; Lam K. S. Nocathiacins, new thiazolyl peptide antibiotics from *Nocardia*sp. II. Isolation, characterization, and structure determination. J. Antibiot. 2003, 56 (3), 232–242. 10.7164/antibiotics.56.232. 12760679

[ref23] Mocek U.; Chen L. C.; Keller P. J.; Houck D. R.; Beale J. M.; Floss H. G. ^1^H and ^13^C NMR assignments of the thiopeptide antibiotic nosiheptide. J. Antibiot. 1989, 42 (11), 1643–1648. 10.7164/antibiotics.42.1643. 2584148

[ref24] Constantine K. L.; Mueller L.; Huang S.; Abid S.; Lam K. S.; Li W.; Leet J. E. Conformation and absolute configuration of nocathiacin I determined by NMR spectroscopy and chiral capillary electrophoresis. J. Am. Chem. Soc. 2002, 124 (25), 7284–7285. 10.1021/ja026249t. 12071733

[ref25] Weber T.; Blin K.; Duddela S.; Krug D.; Kim H. U.; Bruccoleri R.; Lee S. Y.; Fischbach M. A.; Müller R.; Wohlleben W.; Breitling R.; Takano E.; Medema M. H. antiSMASH 3.0-a comprehensive resource for the genome mining of biosynthetic gene clusters. Nucleic Acids Res. 2015, 43 (W1), W237–W243. 10.1093/nar/gkv437. 25948579 PMC4489286

[ref26] Zhang Q.; Li Y.; Chen D.; Yu Y.; Duan L.; Shen B.; Liu W. Radical-mediated enzymatic carbon chain fragmentation-recombination. Nat. Chem. Biol. 2011, 7 (3), 154–160. 10.1038/nchembio.512. 21240261 PMC3079562

[ref27] Nicolet Y.; Zeppieri L.; Amara P.; Fontecilla-Camps J. C. Crystal structure of tryptophan lyase (NosL): evidence for radical formation at the amino group of tryptophan. Angew. Chem., Int. Ed. 2014, 53 (44), 11840–11844. 10.1002/anie.201407320. 25196319

[ref28] Ji X.; Li Y.; Ding W.; Zhang Q. Substrate-Tuned Catalysis of the Radical S-Adenosyl-L-Methionine Enzyme NosL Involved in Nosiheptide Biosynthesis. Angew. Chem., Int. Ed. 2015, 54 (31), 9021–9024. 10.1002/anie.201503976. 26138750

[ref29] Bhandari D. M.; Xu H.; Nicolet Y.; Fontecilla-Camps J. C.; Begley T. P. Tryptophan Lyase (NosL): Mechanistic Insights from Substrate Analogues and Mutagenesis. Biochem 2015, 54 (31), 4767–4769. 10.1021/acs.biochem.5b00764. 26204056

[ref30] Ji X.; Li Y.; Jia Y.; Ding W.; Zhang Q. Mechanistic Insights into the Radical S-adenosyl-l-methionine Enzyme NosL From a Substrate Analogue and the Shunt Products. Angew. Chem., Int. Ed. 2016, 55 (10), 3334–3337. 10.1002/anie.201509900. 26837062

[ref31] Sicoli G.; Mouesca J.-M.; Zeppieri L.; Amara P.; Martin L.; Barra A. L.; Fontecilla-Camps J. C.; Gambarelli S.; Nicolet Y. Fine-tuning of a radical-based reaction by radical S-adenosyl-L-methionine tryptophan lyase. Science 2016, 351 (6279), 1320–1323. 10.1126/science.aad8995. 26989252

[ref32] Ding W.; Ji X.; Li Y.; Zhang Q. Catalytic Promiscuity of the Radical S-adenosyl-L-methionine Enzyme NosL. Front. Chem. 2016, 4 (27), 27 10.3389/fchem.2016.00027. 27446906 PMC4916742

[ref33] Bhandari D. M.; Fedoseyenko D.; Begley T. P. Tryptophan Lyase (NosL): A Cornucopia of 5′-Deoxyadenosyl Radical Mediated Transformations. J. Am. Chem. Soc. 2016, 138 (50), 16184–16187. 10.1021/jacs.6b06139. 27998091

[ref34] Qianzhu H.; Ji W.; Ji X.; Chu L.; Guo C.; Lu W.; Ding W.; Gao J.; Zhang Q. Reactivity of the nitrogen-centered tryptophanyl radical in the catalysis by the radical SAM enzyme NosL. Chem. Commun. 2017, 53 (2), 344–347. 10.1039/C6CC08869D. 27929146

[ref35] Badding E. D.; Grove T. L.; Gadsby L. K.; LaMattina J. W.; Boal A. K.; Booker S. J. Rerouting the Pathway for the Biosynthesis of the Side Ring System of Nosiheptide: The Roles of NosI, NosJ, and NosK. J. Am. Chem. Soc. 2017, 139 (16), 5896–5905. 10.1021/jacs.7b01497. 28343381 PMC5940322

[ref36] Ding W.; Ji W.; Wu Y.; Wu R.; Liu W.-Q.; Mo T.; Zhao J.; Ma X.; Zhang W.; Xu P.; Deng Z.; Tang B.; Yu Y.; Zhang Q. Biosynthesis of the nosiheptide indole side ring centers on a cryptic carrier protein NosJ. Nat. Commun. 2017, 8 (1), 437 10.1038/s41467-017-00439-1. 28874663 PMC5585349

[ref37] Qiu Y.; Du Y.; Zhang F.; Liao R.; Zhou S.; Peng C.; Guo Y.; Liu W. Thiolation Protein-Based Transfer of Indolyl to a Ribosomally Synthesized Polythiazolyl Peptide Intermediate during the Biosynthesis of the Side-Ring System of Nosiheptide. J. Am. Chem. Soc. 2017, 139 (50), 18186–18189. 10.1021/jacs.7b11367. 29200275

[ref38] LaMattina J. W.; Wang B.; Badding E. D.; Gadsby L. K.; Grove T. L.; Booker S. J. NosN, a Radical S-Adenosylmethionine Methylase, Catalyzes Both C1 Transfer and Formation of the Ester Linkage of the Side-Ring System during the Biosynthesis of Nosiheptide. J. Am. Chem. Soc. 2017, 139 (48), 17438–17445. 10.1021/jacs.7b08492. 29039940 PMC5938625

[ref39] Yu Y.; Duan L.; Zhang Q.; Liao R.; Ding Y.; Pan H.; Wendt-Pienkowski E.; Tang G.; Shen B.; Liu W. Nosiheptide biosynthesis featuring a unique indole side ring formation on the characteristic thiopeptide framework. ACS Chem. Biol. 2009, 4 (10), 855–864. 10.1021/cb900133x. 19678698 PMC2763056

[ref40] Ding Y.; Yu Y.; Pan H.; Guo H.; Li Y.; Liu W. Moving posttranslational modifications forward to biosynthesize the glycosylated thiopeptide nocathiacin I in *Nocardia* sp. ATCC202099. Mol. Biosyst. 2010, 6 (7), 1180–1185. 10.1039/c005121g. 20473441

[ref41] Bai X.; Guo H.; Chen D.; Yang Q.; Tao J.; Liu W. Isolation and structure determination of two new nosiheptide-type compounds provide insights into the function of the cytochrome P450 oxygenase NocV in nocathiacin biosynthesis. Org. Chem. Front. 2020, 7 (3), 584–589. 10.1039/C9QO01328H.

[ref42] Guo H.; Bai X.; Yang Q.; Xue Y.; Chen D.; Tao J.; Liu W. NocU is a cytochrome P450 oxygenase catalyzing N-hydroxylation of the indolic moiety during the maturation of the thiopeptide antibiotics nocathiacins. Org. Biomol. Chem. 2021, 19 (38), 8338–8342. 10.1039/D1OB01284C. 34523664

[ref43] Yu Y.; Guo H.; Zhang Q.; Duan L.; Ding Y.; Liao R.; Lei C.; Shen B.; Liu W. NosA catalyzing carboxyl-terminal amide formation in nosiheptide maturation via an enamine dealkylation on the serine-extended precursor peptide. J. Am. Chem. Soc. 2010, 132 (46), 16324–16326. 10.1021/ja106571g. 21047073 PMC2990472

[ref44] Wagstaff B. A.; Zorzoli A.; Dorfmueller H. C. NDP-rhamnose biosynthesis and rhamnosyltransferases: building diverse glycoconjugates in nature. Biochem. J. 2021, 478 (4), 685–701. 10.1042/BCJ20200505. 33599745

[ref45] Thibodeaux C. J.; Melançon C. E.; Liu H.-W. Natural-product sugar biosynthesis and enzymatic glycodiversification. Angew. Chem., Int. Ed. 2008, 47 (51), 9814–9859. 10.1002/anie.200801204. PMC2796923 19058170

[ref46] Du Y.; Xia Y.; Wu L.; Chen L.; Rong J.; Fan J.; Chen Y.; Wu X. Selective biosynthesis of a rhamnosyl nosiheptide by a novel bacterial rhamnosyltransferase. Microb. Biotechnol. 2024, 17 (1), e14412 10.1111/1751-7915.14412. 38265165 PMC10832541

[ref47] Boucher H. W.; Talbot G. H.; Bradley J. S.; Edwards J. E.; Gilbert D.; Rice L. B.; Scheld M.; Spellberg B.; Bartlett J. Bad Bugs, No Drugs: No ESKAPE! An Update from the Infectious Diseases Society of America. Clin. Infect. Dis. 2009, 48 (1), 1–12. 10.1086/595011. 19035777

[ref48] Pucci M. J.; Bronson J. J.; Barrett J. F.; DenBleyker K. L.; Discotto L. F.; Fung-Tomc J. C.; Ueda Y. Antimicrobial Evaluation of Nocathiacins, a Thiazole Peptide Class of Antibiotics. Antimicrob. Agents Chemother. 2004, 48 (10), 3697–3701. 10.1128/aac.48.10.3697-3701.2004. 15388422 PMC521901

[ref49] Singh S. B.; Xu L.; Meinke P. T.; Kurepina N.; Kreiswirth B. N.; Olsen D. B.; Young K. Thiazomycin, nocathiacin and analogs show strong activity against clinical strains of drug-resistant *Mycobacterium tuberculosis*. J. Antibiot. 2017, 70 (5), 671–674. 10.1038/ja.2016.165. 28096545

[ref50] Martin A.; Camacho M.; Portaels F.; Palomino J. C. Resazurin microtiter assay plate testing of *Mycobacterium tuberculosis* susceptibilities to second-line drugs: rapid, simple, and inexpensive method. Antimicrob. Agents Chemother. 2003, 47 (11), 3616–3619. 10.1128/AAC.47.11.3616-3619.2003. 14576129 PMC253784

[ref51] Baumann S.; Schoof S.; Harkal S. D.; Arndt H. D. Mapping the binding site of thiopeptide antibiotics by proximity-induced covalent capture. J. Am. Chem. Soc. 2008, 130 (17), 5664–5666. 10.1021/ja710608w. 18380436

[ref52] Harms J. M.; Wilson D. N.; Schluenzen F.; Connell S. R.; Stachelhaus T.; Zaborowska Z.; Spahn C. M.; Fucini P. Translational regulation via L11: molecular switches on the ribosome turned on and off by thiostrepton and micrococcin. Mol. Cell 2008, 30 (1), 26–38. 10.1016/j.molcel.2008.01.009. 18406324

[ref53] Walter J. D.; Hunter M.; Cobb M.; Traeger G.; Spiegel P. C. Thiostrepton inhibits stable 70S ribosome binding and ribosome-dependent GTPase activation of elongation factor G and elongation factor 4. Nucleic Acids Res. 2012, 40 (1), 360–370. 10.1093/nar/gkr623. 21908407 PMC3245911

[ref54] Woodgate J.; Salliss M. E.; Sumang F. A.; Belousoff M.; Ward A.; Challis G. L.; Zenkin N.; Errington J.; Dashti Y. Mode of Action and Mechanisms of Resistance to the Unusual Polyglycosylated Thiopeptide Antibiotic Persiathiacin A. ACS Infect. Dis. submitted for publication. 10.1021/acsinfecdis.4c00503 PMC11731312 39651842

[ref55] Reasoner D. J.; Geldreich E. E. A new medium for the enumeration and subculture of bacteria from potable water. Appl. Environ. Microbiol. 1985, 49 (1), 1–7. 10.1128/aem.49.1.1-7.1985. 3883894 PMC238333

[ref56] Yoon S.-H.; Ha S.-M.; Kwon S.; Lim J.; Kim Y.; Seo H.; Chun J. Introducing EzBioCloud: a taxonomically united database of 16S rRNA gene sequences and whole-genome assemblies. Int. J. Syst. Evol. Microbiol. 2017, 67 (5), 1613–1617. 10.1099/ijsem.0.001755. 28005526 PMC5563544

[ref57] Li H.; Durbin R. Fast and accurate short read alignment with Burrows-Wheeler transform. Bioinformatics 2009, 25 (14), 1754–1760. 10.1093/bioinformatics/btp324. 19451168 PMC2705234

[ref58] Koboldt D. C.; Zhang Q.; Larson D. E.; Shen D.; McLellan M. D.; Lin L.; Miller C. A.; Mardis E. R.; Ding L.; Wilson R. K. VarScan 2: somatic mutation and copy number alteration discovery in cancer by exome sequencing. Genome Res. 2012, 22 (3), 568–576. 10.1101/gr.129684.111. 22300766 PMC3290792

[ref59] Seemann T. Prokka: rapid prokaryotic genome annotation. Bioinformatics 2014, 30 (14), 2068–2069. 10.1093/bioinformatics/btu153. 24642063

