# Supplementary material for: Discovery and Biosynthesis of Persiathiacins: Unusual Polyglycosylated Thiopeptides Active Against Multidrug Resistant Tuberculosis
Source: ACS Infect Dis. 2024 Aug 27;10(9):3378–91. doi: 10.1021/acsinfecdis.4c00502 (PMC11406533; doi:10.1021/acsinfecdis.4c00502)
Supplement: Supplementary file 1 — id4c00502_si_001.pdf [file id4c00502_si_001.pdf]

Discovery and biosynthesis of persiathiacins: Unusual polyglycosylated thiopeptides active against multi-drug resistant tuberculosis

Yousef Dashti,[ab*] Fatemeh Mohammadipanah,[c] Yu Zhang,[a] Pietra M. Cerqueira Diaz,[a] Anthony Vocat,[d] Daniel Zabala,[a‡] Christopher D. Fage,[a‡] Isolda Romero-Canelon,[ae] Boyke Bunk,[f] Cathrin Spröer,[f] Lona M. Alkhalaf,[a] Jörg Overmann,[fg] Stewart T. Cole [d‡] and Gregory L. Challis [ahij*]

[a] Department of Chemistry, University of Warwick, Coventry CV4 7AL, United Kingdom
[b] Sydney Infectious Diseases Institute, Faculty of Medicine and Health, University of Sydney, Sydney NSW 2015, Australia
[c] Department of Microbiology, School of Biology and Center of Excellence in Phylogeny of Living Organisms, College of Science, University of Tehran, 14155-6455, Tehran, Iran
[d] Global Health Institute, Ecole Polytechnique Fédérale de Lausanne, Station 19, 1015 Lausanne, Switzerland
[e] School of Pharmacy, Institute of Clinical Sciences, University of Birmingham, Birmingham B15 2TT, United Kingdom
[f] Leibniz-Institute DSMZ-German Collection of Microorganisms and Cell Cultures, 38124 Braunschweig, Germany, and Technical University of Braunschweig, 38106 Braunschweig, Germany
[g] German Centre of Infection Research (DZIF), Partner Site Hannover-Braunschweig, 38124 Braunschweig, Germany
[h] Warwick Integrative Synthetic Biology Centre, University of Warwick, Coventry CV4 7AL, United Kingdom
[i] Department of Biochemistry and Molecular Biology, Monash University, Clayton VIC 3168, Australia
[j] ARC Centre of Excellence for Innovations in Peptide and Protein Science, Monash University, Clayton VIC 3168, Australia
[‡] Current addresses: S.T.C. Institut Pasteur, 25 – 28 rue du Docteur Roux, 75015 Paris, France; C.D.F. Institute for Integrative Biology of the Cell (I2BC), CEA, CNRS, Université Paris-Saclay, 91198 Gif-sur-Yvette, France; D.Z. Faes Farma, Autonomia Etorbidea, 10, 48940 Leioa, Bizkaia, Spain.
[*] Corresponding authors: yousef.dashti@sydney.edu.au and g.l.challis@warwick.ac.uk

Contents	Page
Figure S1. Structure of persiathiacin A, numbered as in Table S1.	S2
Table S1. ^{1}H and ^{13}C NMR chemical shifts of persiathiacin A in $CDCl_3$-CD_3OD (9:1).	S3-S5
Figure S2. Structure of persiathiacin B, numbered as in Table S2.	S6
Table S2. ^{1}H and ^{13}C NMR chemical shifts of persiathiacin B in $CDCl_3$-CD_3OD (9:1).	S7-S9
Figure S3. ^{1}H NMR spectrum of persiathiacin A in $CDCl_3$-CD_3OD (9:1).	S10
Figure S4. ^{13}C NMR spectrum of persiathiacin A in $CDCl_3$-CD_3OD (9:1).	S11
Figure S5. ^{1}H-^{1}H COSY spectrum of persiathiacin A in $CDCl_3$-CD_3OD (9:1).	S12
Figure S6. ^{1}H-^{13}C HSQC spectrum of persiathiacin A in $CDCl_3$-CD_3OD (9:1).	S13
Figure S7. ^{1}H-^{13}C HMBC spectrum of persiathiacin A in $CDCl_3$-CD_3OD (9:1).	S14
Figure S8. ^{1}H-^{1}H ROSY spectrum of persiathiacin A in $CDCl_3$-CD_3OD (9:1).	S15
Figure S9. ^{1}H NMR spectrum of persiathiacin B in $CDCl_3$-CD_3OD (9:1).	S16
Figure S10. ^{13}C NMR spectrum of persiathiacin B in $CDCl_3$-CD_3OD (9:1).	S17
Figure S11. ^{1}H-^{1}H COSY spectrum of persiathiacin B in $CDCl_3$-CD_3OD (9:1).	S18
Figure S12. ^{1}H-^{13}C HSQC spectrum of persiathiacin B in $CDCl_3$-CD_3OD (9:1).	S19
Figure S13. ^{1}H-^{13}C HMBC spectrum of persiathiacin B in $CDCl_3$-CD_3OD (9:1).	S20
Figure S14. ^{1}H-^{1}H ROSY spectrum of persiathiacin B in $CDCl_3$-CD_3OD (9:1).	S21
Table S3: Predicted specialized metabolite biosynthetic gene clusters in the genome of *Actinokineospora* sp. UTMC 2448.	S22
Table S4: Genes/proteins in the persiathiacin biosynthetic gene cluster and their similarity to homologous proteins.	S23-S24
Figure S15. Similarities and differences in the activation and attachment of MIA to the nosiheptide, persiathiacin, and nocathiacin processed core peptides.	S25
Figure S16. Mass spectrum of PerX.	S26
Figure S17. SDS-PAGE and intact protein MS of purified recombinant PerS4.	S27
Figure S18. Percentage of A2780 ovarian cancer cells surviving after exposure to increasing concentrations of persiathiacin A (10 to 400 μM) relative to an untreated control.	S27

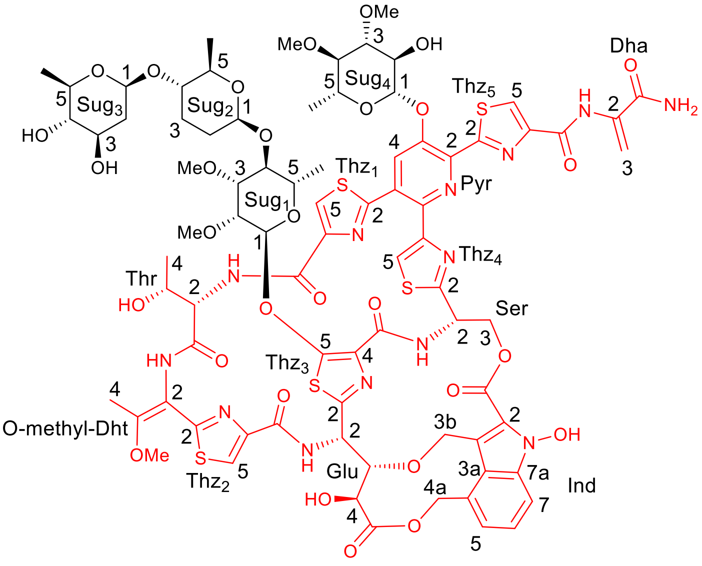

persiathiacin A

Figure S1. Structure of persiathiacin A, numbered as in Table S1.

Table S1. ^{1}H (500 MHz) and ^{13}C (125 MHz) and HMBC NMR data for persiathiacin A in $CDCl_3$-CD_3OD (9:1).

Position		δ_C	δ_H	HMBC	ROESY
Dha	C=O	166.5			
	C2	133.5			
	C3	104.8	H3a: 5.5, brs<br>H3b: 6.5, brs	Dha-(C=O)<br>Dha-(C2, C=O)	Dha-(H3b)<br>Dha-(H3a)
	NH		9.98, s	Dha-(C3, C=O), Thz5-(C4, C=O)	
Thz5	C2	166.9			
	C4	151.2			
	C5	127.2	8.15, s	Thz5-(C2, C4, C=O)	
	C=O	160.0			
Pyr	C2	139.8			
	C3	149.0			
	C4	126.7	7.77, s	Pyr-(C2, C3, C6), Thz-(C2)	Sug4-(H1)
	C5	128.3			
	C6	144.8			
Thz1	C2	165.5			
	C4	149.1			
	C5	125.2	8.25, s	Thz1-(C2, C4, C=O)	Thr-(H4)
	C=O	161.8			
Thr	NH		7.87, brs	Thz1-(C=O)	
	C=O	167.5			
	C2	56.2	4.21, dd, J = 4.0, 5.5	Thr-(C3, C4, C=O), Thz1-(C=O)	Thr-(H3)<br>Dht-(NH)
	C3	64.7	2.88, m	Thr-(C=O)	Thr-(H2)
	C4	17.7	1.34, d, J = 6.0	Thr-(C2, C3)	Thz1-(H5)
O-methyl-Dht	NH		8.31, s	Dht-(C3), Thr-(C=O)	Thr-(H2)
	C2	110.7			
	C3	158.7			
	C4	13.7	1.86, s	Dht-(C2, C3), Thz2-(C2)	Dht-(NH, OCH_3), Thr-(H2)
	OMe	55.8	3.78, s	Dht-(C3)	Dht-(H4)
Thz2	C2	161.8			
	C4	146.1			
	C5	124.3	7.96, s	Thz2-(C2, C4, C=O), Dht-(C2)	
	C=O	161.7			
Glu	NH		8.24, brs	Glu-(C2, C3), Thz2-(C=O)	Glu-(H2, H4)
	C2	48.7	5.80, d, J = 10.0	Glu-(C3, C4), Thz3-(C2)	Glu-(NH, H3, H4)
	C3	82.9	3.65, m	Glu-(C4, C=O), Thz3-(C2), Ind (C3b)	Glu-(H2), Ind-(H3ba/b)

	C4	67.7	4.09, d, *J* = 10.0	Glu-(C2, C3, C=O)	Glu-(NH, H2, H3)
	C=O	174.5			
Thz3	C2	154.8			
	C4	130.9			
	C5	160.3			
	C=O	160.3			
Ser	NH		7.89, d, *J* = 11.0	Ser-(C2, C3), Thz3-(C4, C=O)	Ser-(H2, H3b)
	C2	48.6	5.62, dd, *J* = 6.0, 10.5	Ser-(C3), Thz3-(C=O), Thz4-(C2)	Ser-(NH, H3a/b)
	C3	64.5	H3a: 4.36, d, *J* = 11.5<br>H3b: 5.21, dd, *J* = 6.0, 11.5	Ser-(C2), Thz4-(C2), Ind-(C=O)<br>Ser-(C2), Thz4-(C2), Ind-(C=O)	Ser-(H2, H3b)<br>Ser-(NH, H2, H3a)
Thz4	C2	169.8			
	C4	154.7			
	C5	121.2	7.77, s	Thz4-(C2, C4), Pyr-(C6)	
Ind	C=O	161.4			
	C2	127.0			
	C3	109.8			
	C3a	119.5			
	C3b	65.9	H3ba: 4.17, d, *J* = 10.5<br><br>H3bb: 4.91, d, *J* = 10.5	Ind-(C2, C3, C3a), Glu-(C3)<br>Ind-(C2, C3, C3a)	Glu-(H3), Ind-(H3bb, H4ab)<br>Glu-(H3), Ind-(H3ba), Ser-(NH)
	C4	127.8			
	C4a	68.4	H4aa: 4.95, d, *J* = 12.5<br><br>H4ab: 5.92, d, *J* = 12.5	Ind-(C3a, C4, C5), Glu-(C=O)<br>Ind-(C3a, C4, C5), Glu-(C=O)	Ind-(H3ba, H4ab, H5)<br>Ind-(H3ba, H4aa, H5)
	C5	123.5	7.10, d, *J* = 7.0	Ind-(C3a, C4a, C6, C7)	Ind-(H4aa, H4ab, H6)
	C6	125.1	7.35, dd, *J* = 7.0, 8.5	Ind-(C4, C5, C7, C7a)	Ind-(H5, H7)
	C7	112.1	7.74, d, *J* = 8.5	Ind-(C3a, C5)	Ind-(H6)
	C7a	135.3			
	N-OH		10.46, s	Ind-(C2)	
Sug1	C1	101.9	5.41, brs	Thz3-(C5), Sug1-(C3, C5)	Sug1-(H2, H4, C2-OCH_3)
	C2	76.0	4.00, m	Sug1-(C1, C3, C4, C2-OMe)	Sug1-(H1, H3, C2-OCH_3)
	C2-OMe	59.4	3.48, s	Sug1-(C2)	Sug1-(H1)
	C3	80.1	3.60, m	Sug1-(C1, C5, C3-OMe)	Sug1-(H2, C3-OCH_3)
	C3-OMe	57.9	3.42, s	Sug1-(C3)	Sug1-(H2, H4)
	C4	77.0	3.64, m	Sug1-(C2, C5, C6), Sug2-(C1)	Sug2-(H1)
	C5	70.1	3.63, m	Sug1-(C1, C3, C4, C6)	Sug1-(H2, H6)

	C6	17.7	1.31, d, *J* = 5.5	Sug1-(C4, C5)	Sug1-(H4)
Sug2	C1	102.2	4.61, d, *J* = 9.0	Sug1-(C4), Sug2-(C2, C5)	Sug1-(H4), Sug2-(H2b, H3a, H5)
	C2	30.8	H2a: 1.39, m<br><br>H2b: 1.79, m	Sug2-(C1, C3, C4)	Sug2-(H1, H2b H3b, H4)<br>Sug2-(H1, H2a H3b)
	C3	29.9	H3a: 1.40, m<br>H3b: 2.09, m	Sug2-(C1, C4)	Sug2-(H2b, H4)
	C4	80.6	3.08, m	Sug2-(C5, C6), Sug3-(C1)	Sug3-(H1), Sug2-(H3a/b, H6)
	C5	74.1	3.26, m	Sug2-(C1, C3, C6)	Sug2-(H1, H3b, H6)
	C6	18.0	1.12, d, *J* = 6.0	Sug2-(C4, C5)	Sug2-(H4, H5), Sug3-(H1)
Sug3	C1	101.1	4.43, d, *J* = 9.5	Sug2-(C4), Sug3-(C2, C3, C5)	Sug2-(H4, H6), Sug3-(H2a, H3, H5)
	C2	39.0	H2a: 1.46, m<br>H2b: 2.06, m	Sug3-(C1, C3, C4)	Sug3-(H2b, H4)<br>Sug3-(H1, H2a, H3)
	C3	71.3	3.42, *ol**	Sug3-(C1, C5)	Sug3-(H1, H2b)
	C4	77.0	2.90, m	Sug3-(C2, C5, C6)	Sug3-(H2a, H3, H6)
	C5	71.8	3.14, m	Sug3-(C1, C4, C6)	Sug3-(H1, H3 H6)
	C6	17.7	1.20, d, *J* = 6.0	Sug3-(C4, C5)	Sug3-(H4, H5)
Sug4	C1	100.4	5.18, d, *J* = 7.5	Pyr-(C3), Sug4-(C3, C5)	Pyr-(H4), Sug4-(H2, H3, H5)
	C2	69.5	4.14, dd, *J* = 7.5, 10.0	Sug4-(C1, C3)	Sug4-(H1, C3-OCH_3)
	C3	84.2	3.17, *ol*	Sug4-(C2, C4, C3-OMe)	Sug4-(H1, H5)
	C3-OMe	58.1	3.45, s	Sug4-(C3)	
	C4	77.6	3.42, *ol*	Sug4-(C2, C4-OMe)	Sug4-(H6)
	C4-OMe	61.8	3.51, s	Sug4-(C4)	Sug4-(H6)
	C5	71.3	3.76, m	Sug4-(C1, C3, C4, C6)	Sug4-(H1, H4, H6)
	C6	16.7	1.30, d, *J* = 6.0	Sug4-(C4, C5)	

* overlapped

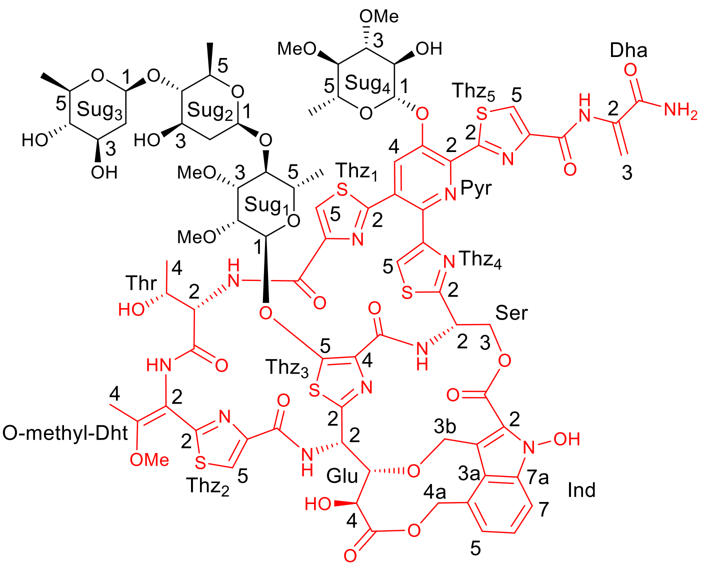

persiathiacin B

Figure S2. Structure of persiathiacin B, numbered as in Table S2.

Table S2. ^{1}H (700 MHz) and ^{13}C (176 MHz) and HMBC NMR data of persiathiacin B in $CDCl_3$-CD_3OD (9:1).

Position		δ_C	δ_H	HMBC	ROESY
Dha	C=O	166.7			
	C2	133.5			
	C3	105.1	H3a: 5.56, brs<br>H3b: 6.51, brs	Dha-(C=O)<br>Dha-(C2, C=O)	Dha-(H3b)<br>Dha-(H3a)
	NH		9.98, s	Deala-(C3, C=O), Thz5-(C4, C=O)	
Thz5	C2	167.1			
	C4	151.2			
	C5	127.4	8.21, s	Thz5-(C2, C4, C=O)	
	C=O	160.1			
Pyr	C2	139.9			
	C3	149.1			
	C4	126.9	7.79, s	Pyr-(C2, C3, C6), Thz-(C2)	Sug4-(H1)
	C5	128.4			
	C6	145.0			
Thz1	C2	165.5			
	C4	149.1			
	C5	125.2	8.27, s	Thz1-(C2, C4, C=O)	Thr-(H4)
	C=O	161.9			
Thr	NH		7.94, brs	Thz1-(C=O)	
	C=O	167.5			
	C2	56.2	4.26, t, *J* = 4.0	Thr-(C3, C4, C=O), Thz1-(C=O)	Thr-(H3)<br>Dht-(NH)
	C3	64.8	2.90, m	Thr-(C=O)	Thr-(H2)
	C4	17.6	1.37, d, *J* = 6.0	Thr-(C2, C3)	Thz1-(H5)
O-methyl-Dht	NH		8.35, s	Dht-(C3), Thr-(C=O)	Thr-(H2)
	C2	110.7			
	C3	158.8			
	C4	13.7	1.89, s	Dht-(C2, C3), Thz2-(C2)	Dht-(NH, OCH_3), Thr-(H2)
	OMe	55.9	3.81, s	Dht-(C3)	Dht-(H4)
Thz2	C2	161.8			
	C4	146.1			
	C5	124.4	7.99, s	Thz2-(C2, C4, C=O), Dht-(C2)	
	C=O	161.8			
Glu	NH		8.27, brs	Glu-(C2, C3), Thz2-(C=O)	Glu-(NH, H2, H4)
	C2	48.7	5.82, dd, *J* = 1.5, 10.0	Glu-(C3, C4), Thz3-(C2)	Glu-(H3, H4)

	C3	83.0	3.66, m	Glu-(C4, C=O), Thz3-(C2), Ind (C3b)	Glu-(H2), Ind-(H3ba/b)
	C4	67.8	4.12, d, *J* = 10.0	Glu-(C2, C3, C=O)	Glu-(NH, H2, H3)
	C=O	174.5			
Thz3	C2	154.7			
	C4	131.0			
	C5	160.3			
	C=O	160.3			
Ser	NH		7.91, d, *J* = 11.0	Ser-(C2, C3), Thz3-(C4, C=O)	Ser-(H2, H3b)
	C2	48.6	5.66, dd, *J* = 6.0, 11.0	Ser-(C3), Thz3-(C=O), Thz4-(C2)	Ser-(NH, H3a/b)
	C3	64.4	H3a: 4.40, d, *J* = 11.5<br>H3b: 5.25, dd, *J* = 6.0, 11.5	Ser-(C2), Thz4-(C2), Ind-(C=O)<br>Ser-(C2), Thz4-(C2), Ind-(C=O)	Ser-(H2, H3b)<br>Ser-(NH, H2, H3a)
Thz4	C2	169.9			
	C4	154.7			
	C5	121.3	7.76, s	Thz4-(C2, C4), Pyr-(C6)	
Ind	C=O	161.4			
	C2	127.1			
	C3	109.8			
	C3a	119.6			
	C3b	65.9	H3ba: 4.22, d, *J* = 10.5<br>H3bb: 4.95, d, *J* = 10.5	Ind-(C2, C3, C3a), Glu-(C3)<br>Ind-(C2, C3, C3a)	Glu-(H3), Ind-(H3bb, H4ab)<br>Glu-(H3), Ind-(H3ba), Ser-(NH)
	C4	127.9			
	C4a	68.4	H4aa: 5.00, d, *J* = 12.5<br>H4ab: 5.97, d, *J* = 12.5	Ind-(C3a, C4, C5), Glu-(C=O)<br>Ind-(C3a, C4, C5), Glu-(C=O)	Ind-(H3ba, H4ab, H5)<br>Ind-(H3ba, H4aa, H5)
	C5	123.6	7.14, d, *J* = 7.0	Ind-(C3a, C4a, C6, C7)	Ind-(H4aa/b, H6)
	C6	125.1	7.39, dd, *J* = 7.0, 8.5	Ind-(C4, C5, C7, C7a)	Ind-(H5, H7)
	C7	112.2	7.80, d, *J* = 8.5	Ind-(C3a, C5)	Ind-(H6)
	C7a	135.3			
	N-OH		10.51, s	Ind-(C2)	
Sug1	C1	101.8	5.45, brs	Thz3-(C5), Sug1-(C2, C3, C5)	Sug1-(H2, H4, C2-OCH_3)
	C2	75.9	4.04, m	Sug1-(C1, C3, C4, C2-OMe)	Sug1-(H1, H3, C2-OCH_3)
	C2-OMe	59.4	3.51, s	Sug1-(C2)	Sug1-(H1, H2)
	C3	80.1	3.64, m	Sug1-(C4, C3-OMe)	Sug1-(H2, C3-OCH_3)

	C3-OMe	57.9	3.45, s	Sug1-(C3)	Sug1-(H2, H4)
	C4	77.3	3.67, m	Sug1-(C5), Sugar 2-(C1)	Sug1-(H6, C3-OCH_3), Sug2-(H1)
	C5	70.0	3.68, m	Sug1-(C1, C3, C6)	
	C6	17.4	1.34, d, *J* = 5.5	Sug1-(C4, C5)	Sug1-(H4)
Sug2	C1	100.2	4.71, dd, *J* = 1.5, 9.0	Sug1-(C4), Sug2-(C2)	Sug1-(H4), Sug2-(H2b, H3, H5)
	C2	38.3	H2a: 1.41, m<br><br>H2b: 2.20, m	Sug2-(C1, C3, C4)	Sug2-(H2b, H4)<br>Sug2-(H1, H2a, H3)
	C3	69.6	3.52, m	Sug2-(C4)	Sug2-(H1, H2b)
	C4	88.2	2.90, m	Sug2-(C5, C6), Sug3-(C1)	Sug3-(H1), Sug2-(H6)
	C5	70.0	3.23, m	Sug2-(C1, C4, C6)	Sug2-(H1, H3, H6)
	C6	17.6	1.18, d, *J* = 6.0	Sug2-(C4, C5)	Sug2-(H4, H5)
Sug3	C1	101.0	4.45, dd, *J* = 2.0, 10.0	Sug2-(C4), Sug3-(C2, C3, C5)	Sug2-(H4, H6), Sug3-(H2b, H3, H5)
	C2	38.7	H2a: 1.56, m<br><br>H2b: 2.16, m	Sug3-(C1, C3, C4)	Sug3-(H2b, H4)<br>Sug3-(H1, H2b)
	C3	70.9	3.48, *ol*		Sug3-(H1, H2b, H5)
	C4	76.7	2.96, m	Sug3-(C3, C5, C6)	Sug3-(H2a, H6)
	C5	72.3	3.28, m	Sug3-(C1, C3, C4, C6)	Sug3-(H1, H6)
	C6	17.4	1.26, d, *J* = 6.0	Sug3-(C4, C5)	Sug3-(H4, H5)
Sug4	C1	100.5	5.18, d, *J* = 7.5	Pyr-(C3), Sug4-(C3, C5)	Pyr-(H4), Sug4-(H3, H5)
	C2	69.5	4.19, dd, *J* = 7.5, 9.5	Sug4-(C1, C3)	Sug4-(H1, H3)
	C3	84.3	3.24, *ol*	Sug4-(C1, C2, C3-OMe)	Sug4-(H1, H2, H5)
	C3-OMe	58.1	3.50, s	Sug4-(C3)	Sug4-(H3)
	C4	77.6	3.48, *ol*	Sug4-(C4-OMe)	Sug4-(H5, H6)
	C4-OMe	61.9	3.56, s	Sug4-(C4)	Sug4-(H6)
	C5	71.3	3.78, m	Sug4-(C1, C3, C4, C6)	Sug4-(H1, H3, H4, H6)
	C6	16.8	1.35, d, *J* = 6.5	Sug4-(C4, C5)	Sug4-(H4, H5, C4-OCH_3)

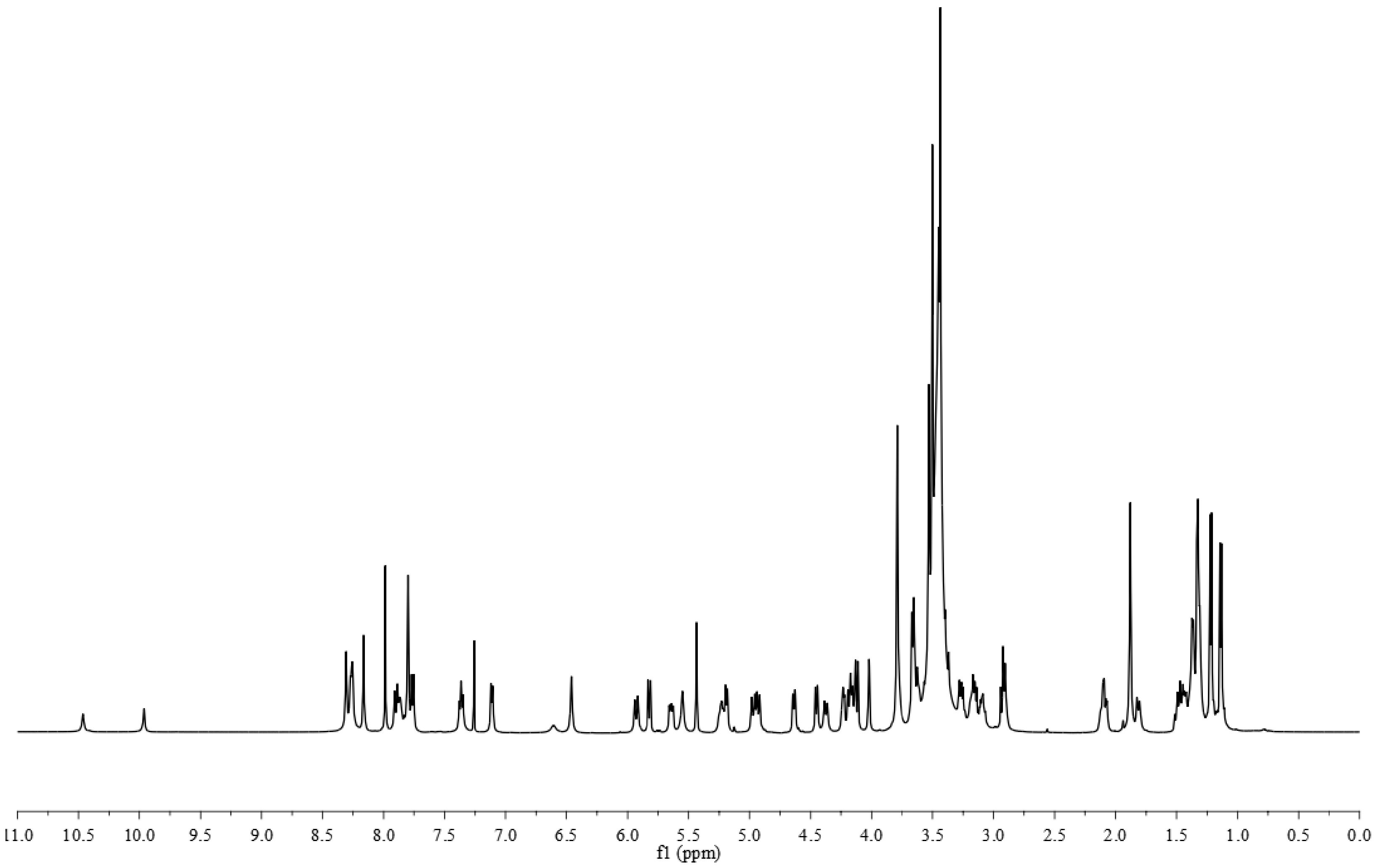

Figure S3. ^{1}H NMR spectrum (500 MHz) of persiathiacin A in $CDCl_3$-CD_3OD (9:1).

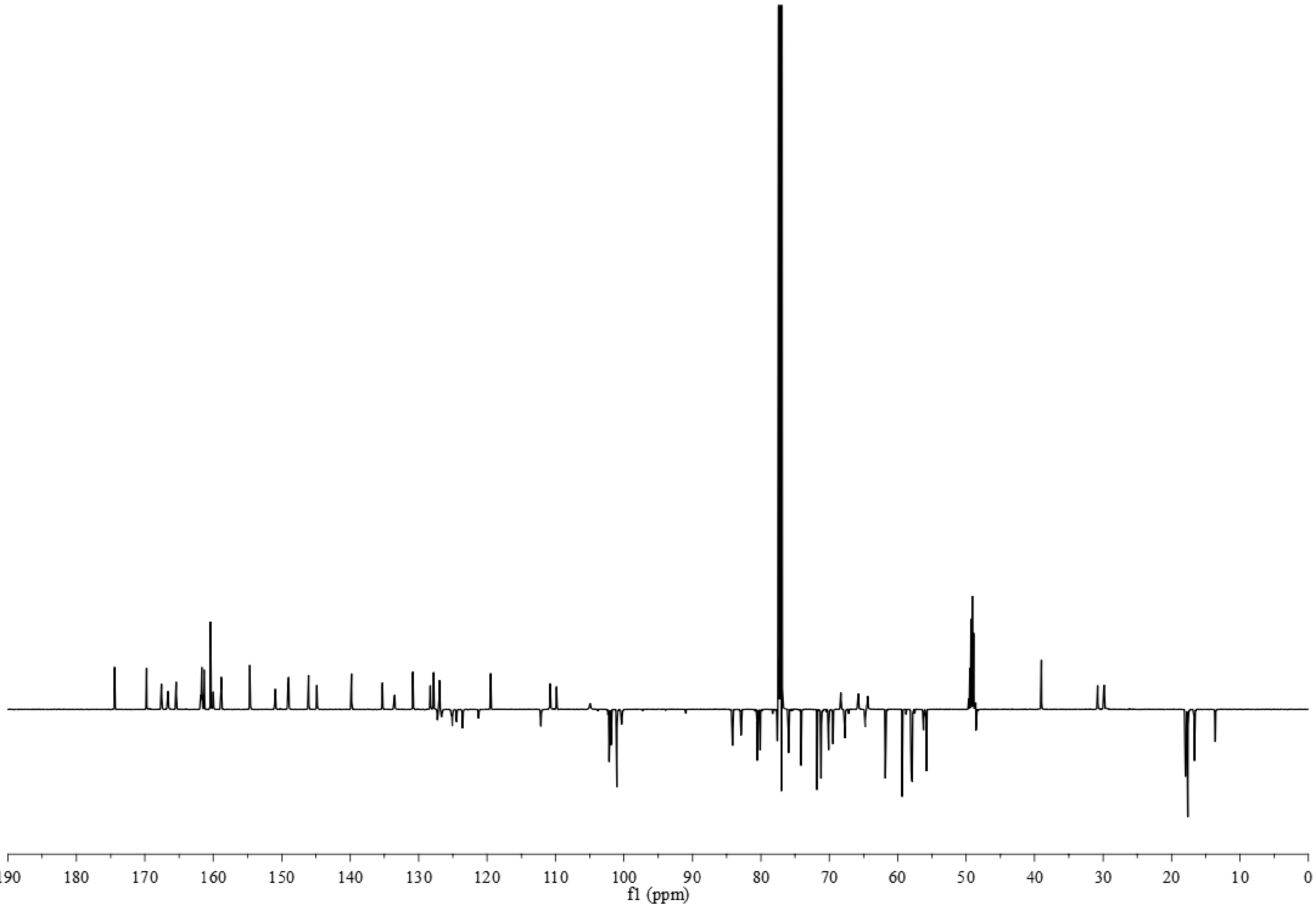

Figure S4. ^{13}C NMR spectrum (125 MHz) of persiathiacin A in $CDCl_3$-CD_3OD (9:1).

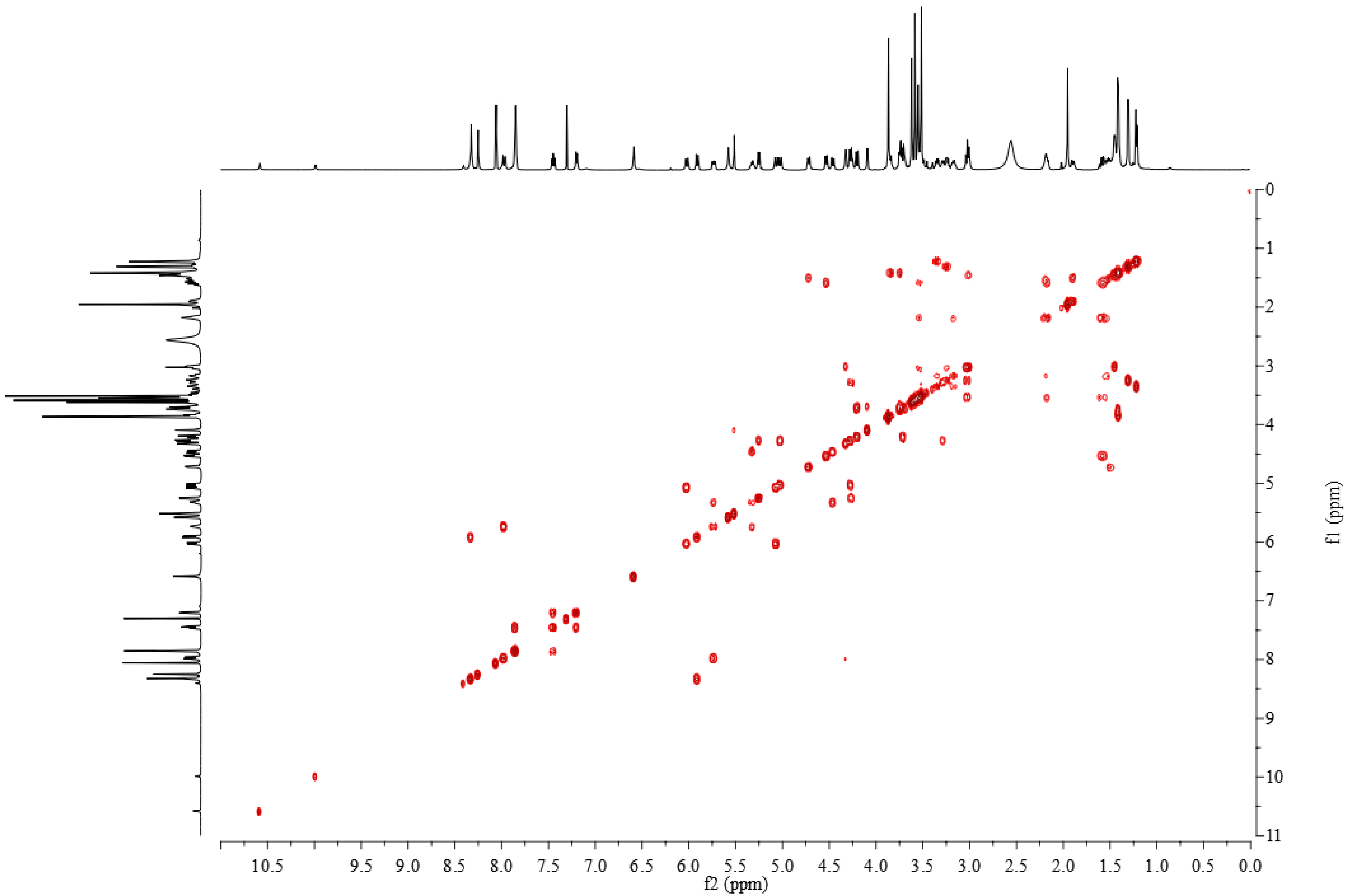

Figure S5. ^{1}H-^{1}H COSY spectrum of persiathiacin A in $CDCl_3$-CD_3OD (9:1).

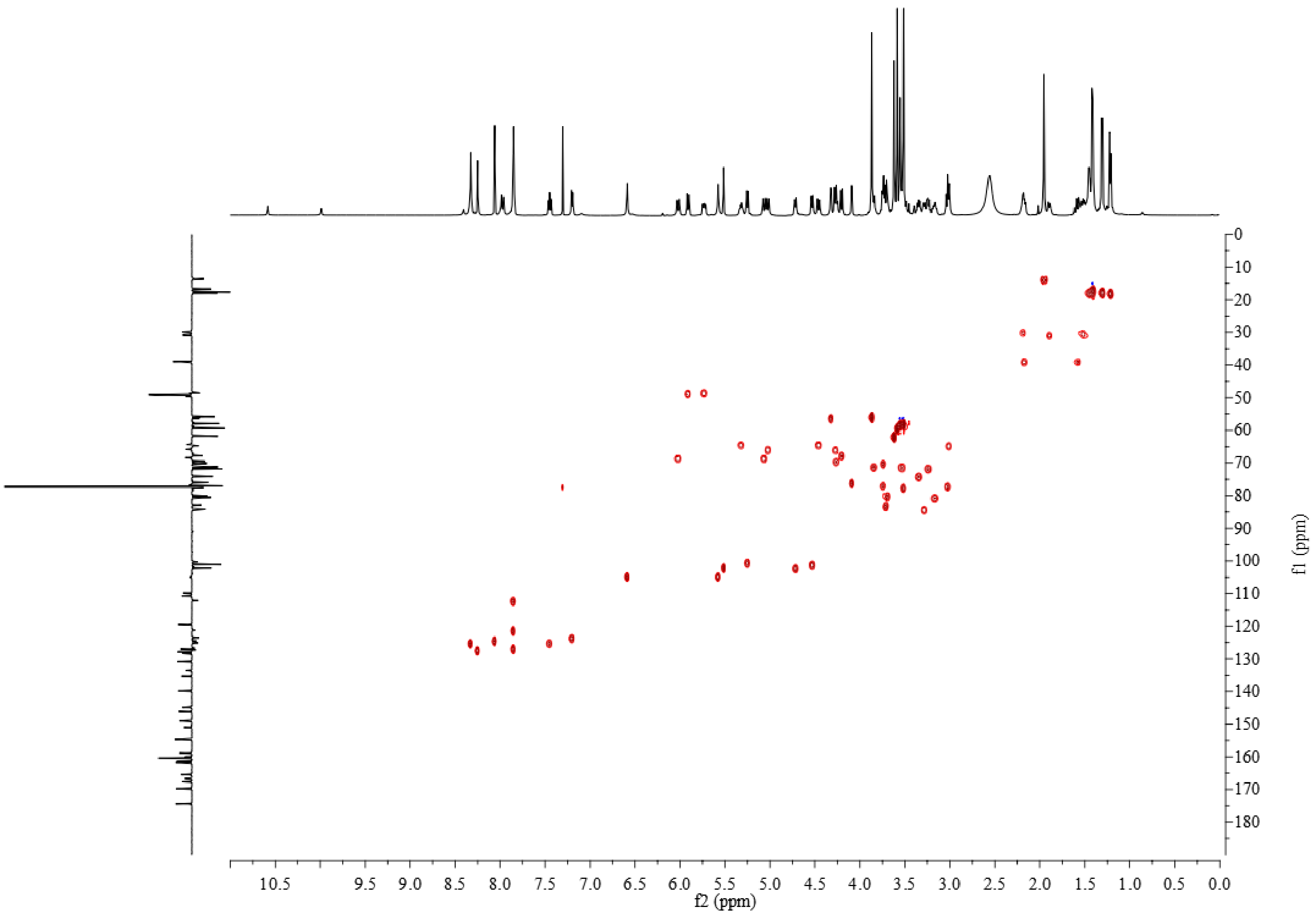

Figure S6. ^{1}H-^{13}C HSQC spectrum of persiathiacin A in $CDCl_3$-CD_3OD (9:1).

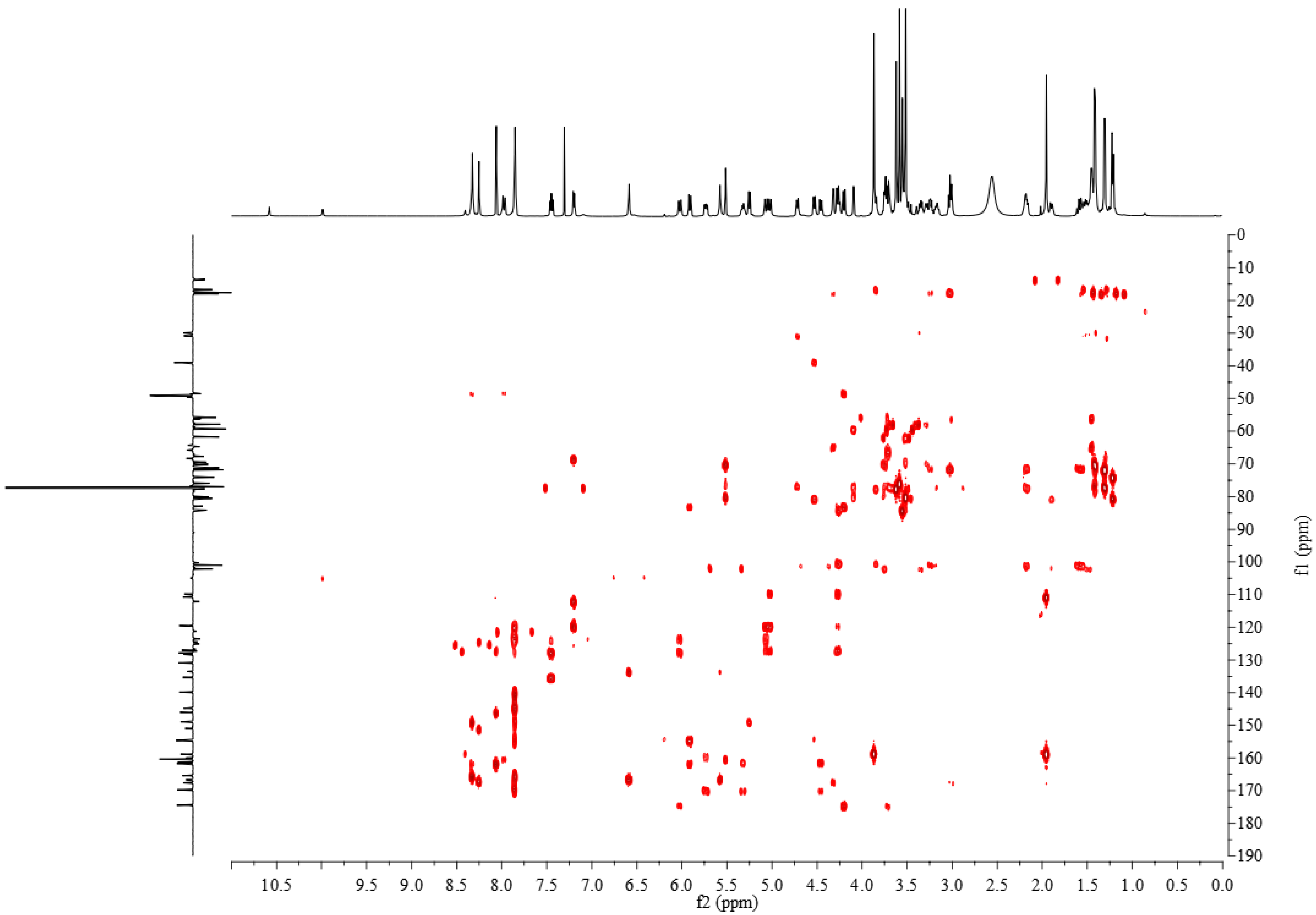

Figure S7. ^{1}H-^{13}C HMBC spectrum of persiathiacin A in $CDCl_3$-CD_3OD (9:1).

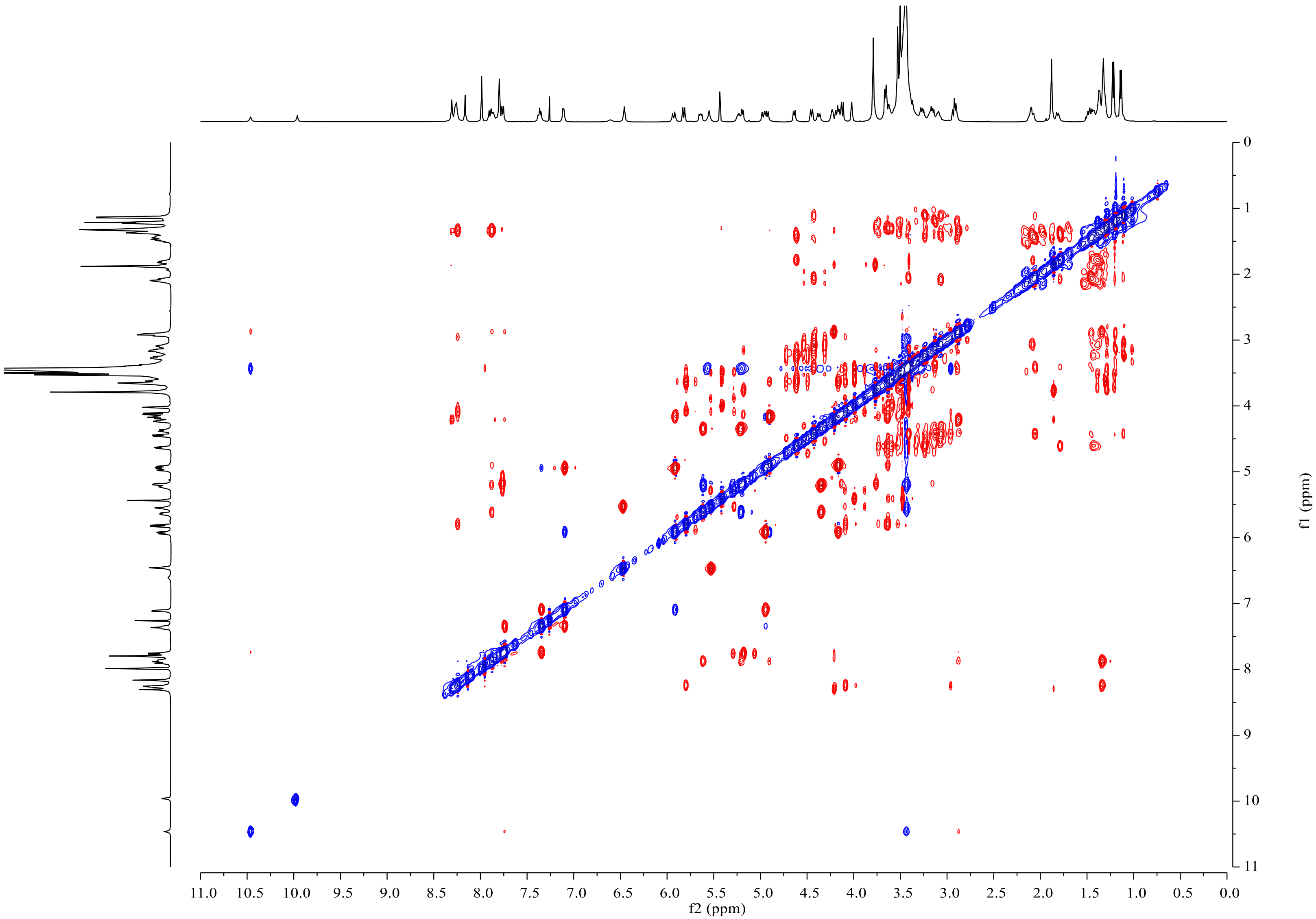

Figure S8. ^{1}H-^{1}H ROESY spectrum of persiathiacin A in $CDCl_3$-CD_3OD (9:1).

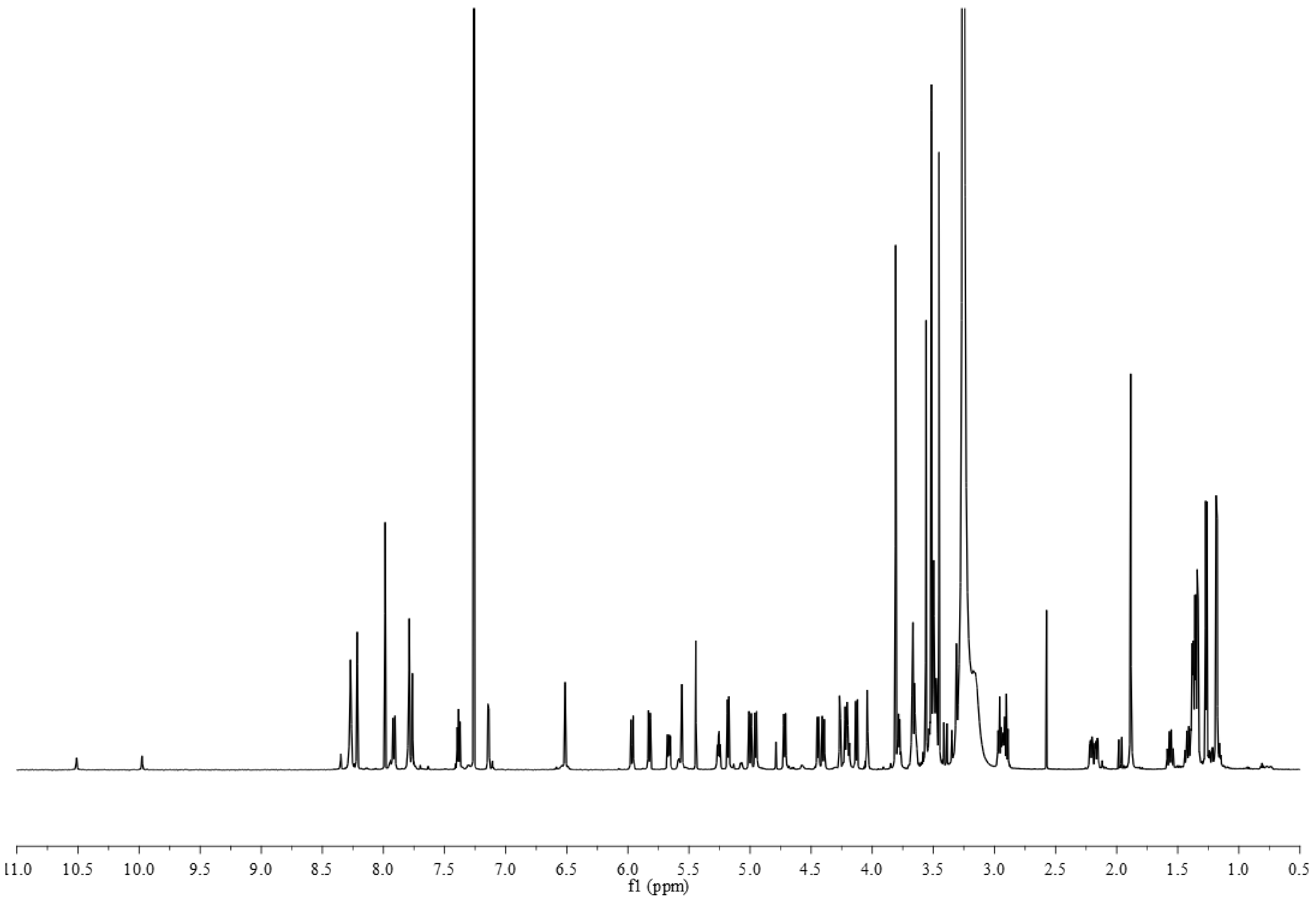

Figure S9. ^{1}H NMR spectrum (700 MHz) of persiathiacin B in $CDCl_3$-CD_3OD (9:1).

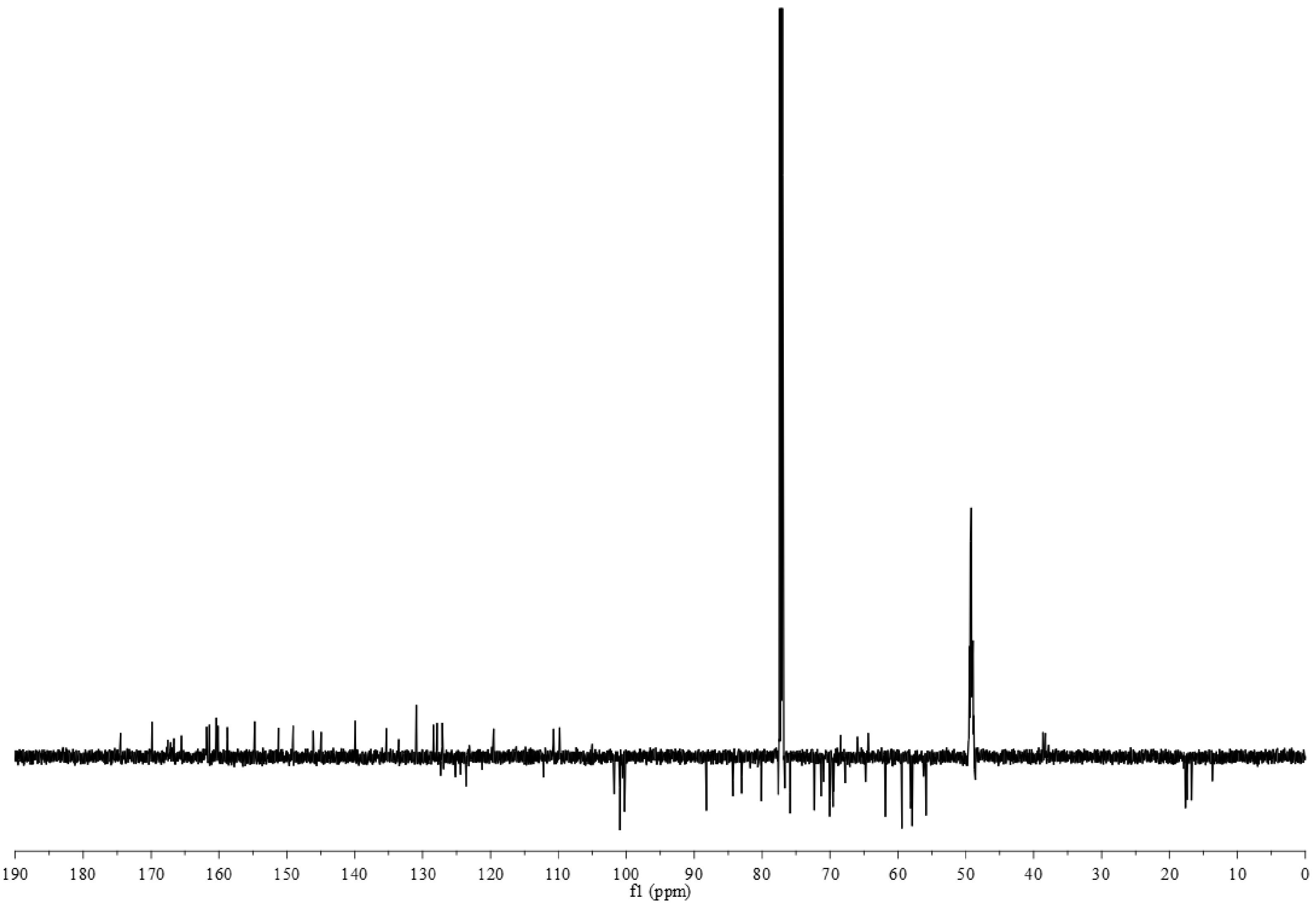

Figure S10. ^{13}C NMR spectrum (176 MHz) of persiathiacin B in $CDCl_3$-CD_3OD (9:1).

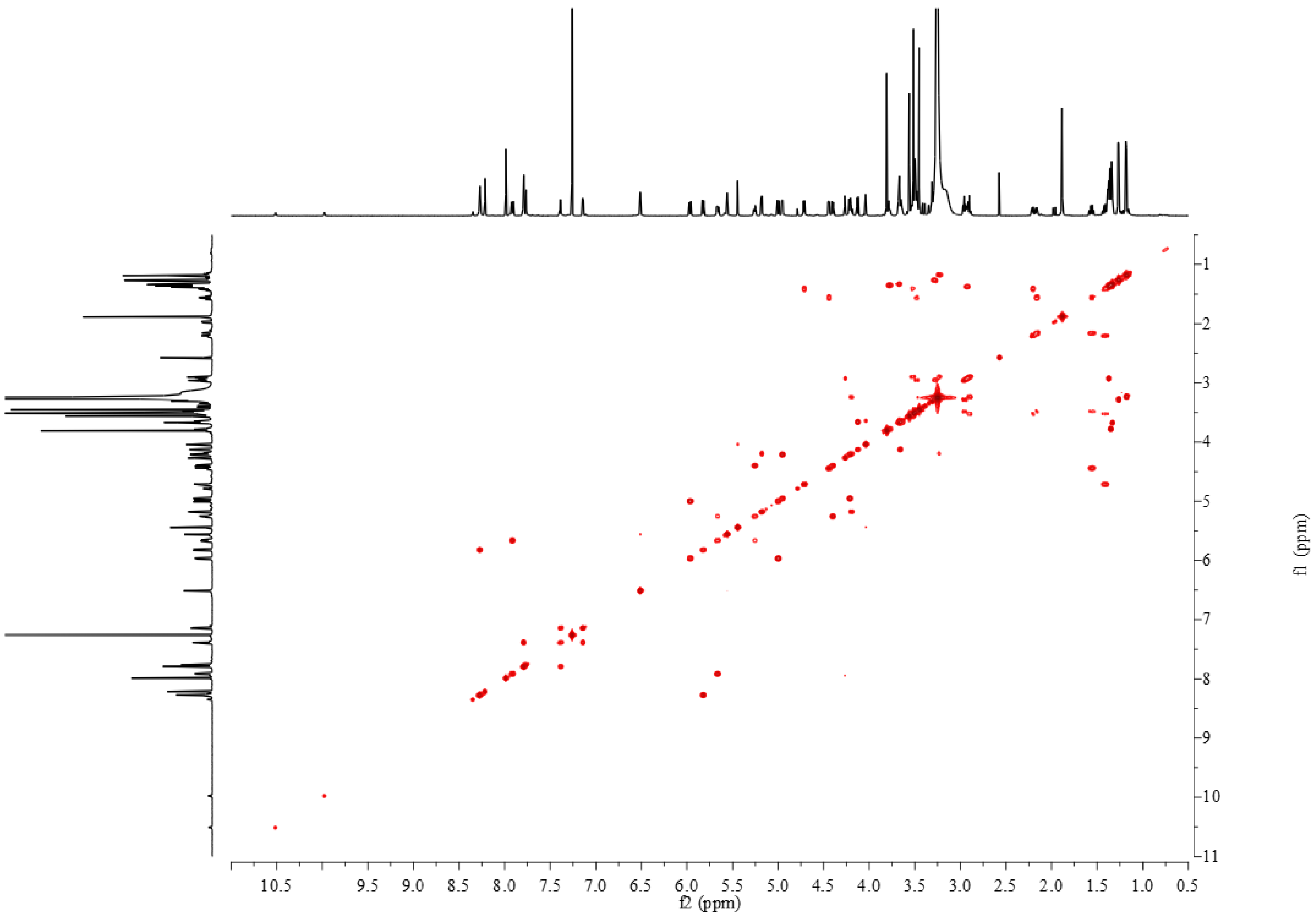

Figure S11. ${}^{1}H$-${}^{1}H$ COSY spectrum of persiathiacin B in $CDCl_3$-CD_3OD (9:1).

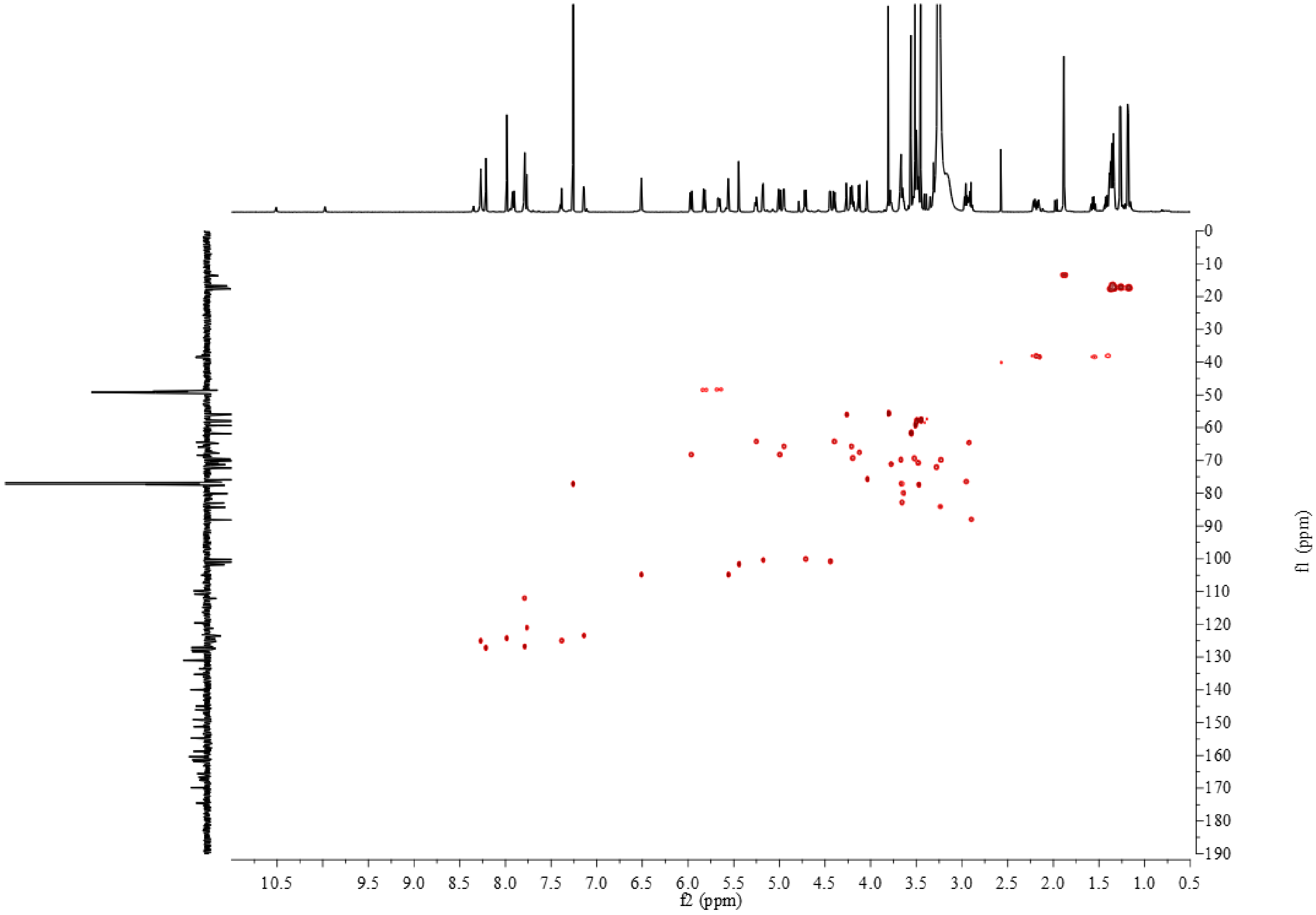

Figure S12. ^{1}H-^{13}C HSQC spectrum of persiathiacin B in $CDCl_3$-CD_3OD (9:1).

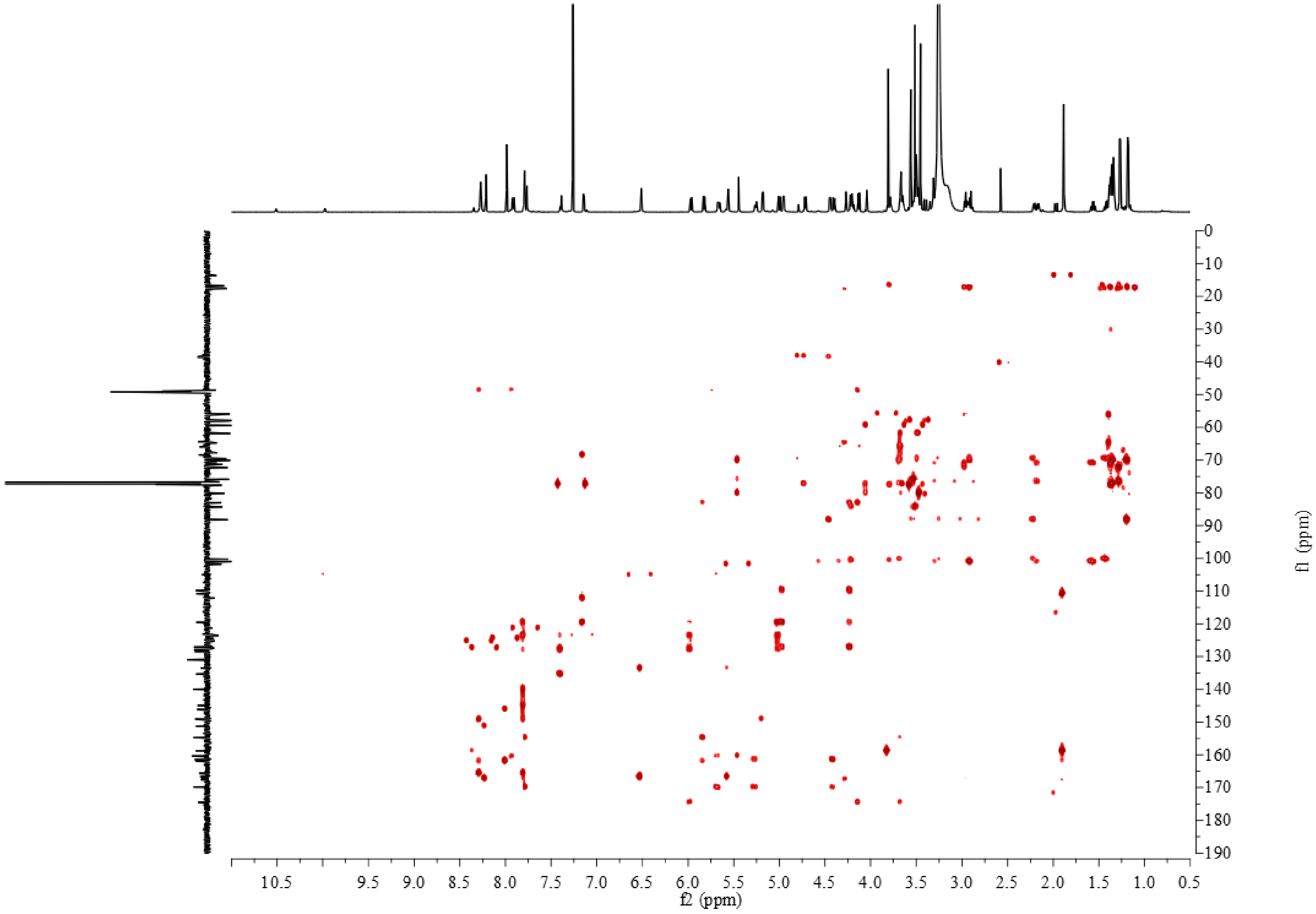

Figure S13. ^{1}H-^{13}C HMBC spectrum of persiathiacin B in $CDCl_3$-CD_3OD (9:1).

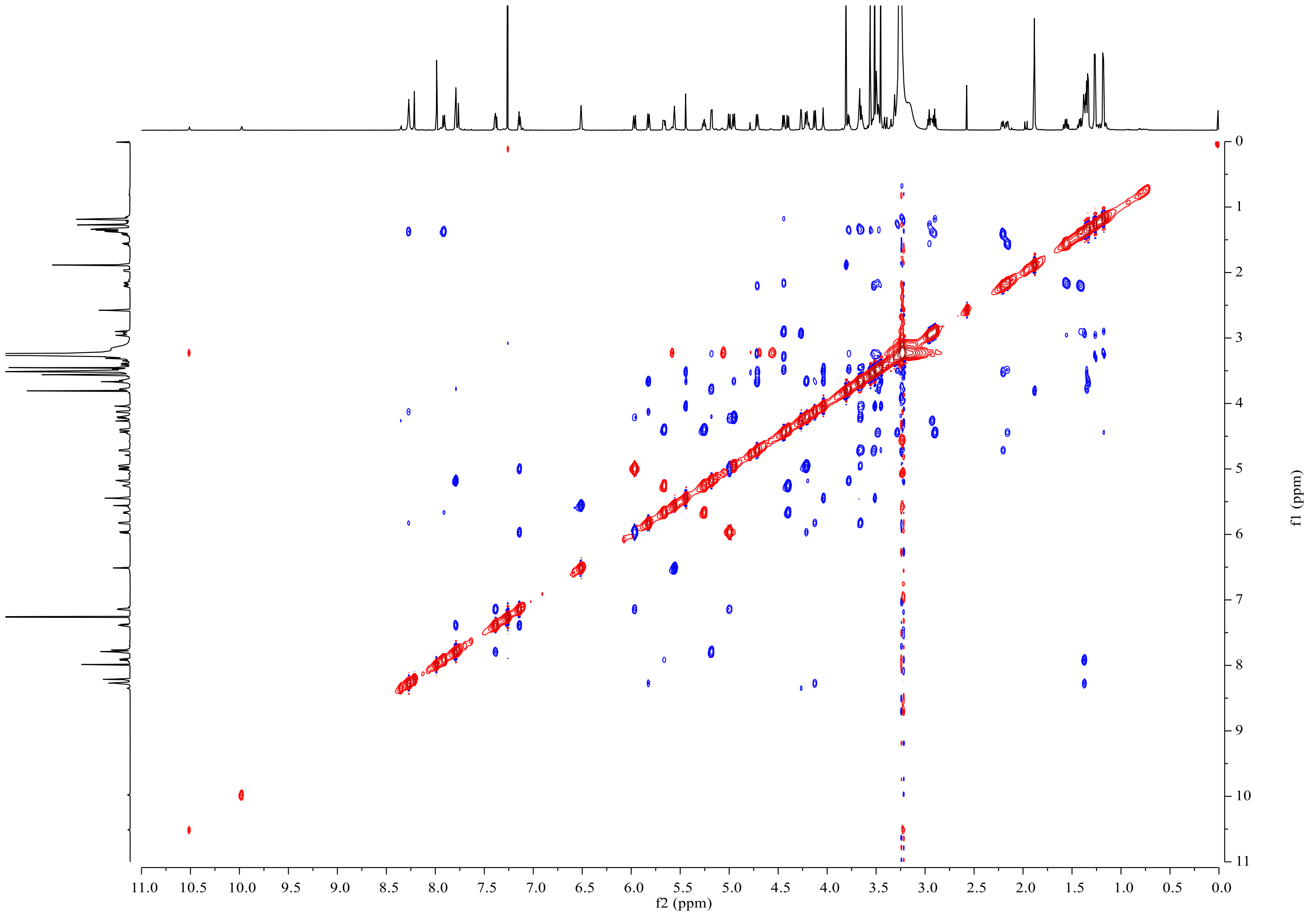

Figure S14. ^{1}H-^{1}H ROESY spectrum of persiathiacin B in $CDCl_3$-CD_3OD (9:1).

Table S3. Predicted specialized metabolite biosynthetic gene clusters in the genome of *Actinokineospora* sp. UTMC 2448.

Cluster	antiSMASH functional prediction	Start position	End position
Cluster 1	Arylpolyene	361740	403998
Cluster 2	Ectoine	427620	438018
Cluster 3	Terpene	557729	606490
Cluster 4	T1pks-Nrps	830346	897979
Cluster 5	Terpene	1663302	1684207
Cluster 6	Bacteriocin	1814304	1825113
Cluster 7	Siderophore	1872946	1884691
Cluster 8	Nrps-Lantipeptide	1895748	1950324
Cluster 9	T1pks	1951254	1997820
Cluster 10	Terpene	2341218	2362180
Cluster 11	Thiopeptide-Oligosaccharide	2544416	2605135
Cluster 12	T2pks	2622827	2665306
Cluster 13	T3pks	2694719	2735756
Cluster 14	Otherks	2799423	2846466
Cluster 15	T1pks	2851965	2898111
Cluster 16	Indole	2927064	2948149
Cluster 17	Phenazine	3024546	3045010
Cluster 18	Nrps	3156780	3256358
Cluster 19	Terpene	3260280	3281989
Cluster 20	Nrps	3330279	3386499
Cluster 21	Ectoine	3571813	3582196
Cluster 22	Butyrolactone-Nrps	3579882	3652648
Cluster 23	T1pks	3904090	3957797
Cluster 24	Other	4140452	4181138
Cluster 25	Terpene	4239895	4261055
Cluster 26	Melanin	4434945	4445863
Cluster 27	T1pks	4577600	4623818
Cluster 28	Lantipeptide	4650662	4674898
Cluster 29	Ladderane-Arylpolyene-Nrps	4820757	4897663
Cluster 30	Lantipeptide-T1pks-NRPS	5700170	5876737
Cluster 31	Bacteriocin	6203188	6214081
Cluster 32	T1pks-Butyrolactone	6252778	6312920

Table S4. Genes in the persiathiacin biosynthetic gene cluster and the percentage identity of the proteins they encode to proteins of known function.

Gene/Protein	Locus_tag	Length bp/aa	Similar Proteins	% aa Identity
perO/PerO	Actin_02399	906/302	NocO (*Nocardia* sp. ATCC202099)<br>NosO (*Streptomyces actuosus*)	52<br>44
perN/PerN	Actin_02400	1236/412	NocN (*Nocardia* sp. ATCC202099)<br>NosN (*Streptomyces actuosus*)	85<br>75
perM/PerM	Actin_02401	147/49	NocM (*Nocardia* sp. ATCC202099)<br>NosM (*Streptomyces actuosus*)	98<br>88
perL/PerL	Actin_02402	1188/396	NocL (*Nocardia* sp. ATCC202099)<br>NosL (*Streptomyces actuosus*)	85<br>82
perK/PerK	Actin_02403	846/282	NocK (*Nocardia* sp. ATCC202099)<br>NosK (*Streptomyces actuosus*)	67<br>59
perI/PerI	Actin_02404	1260/420	NocI (*Nocardia* sp. ATCC202099)<br>NosI (*Streptomyces actuosus*)	67<br>62
perH/PerH	Actin_02405	1728/576	NocH (*Nocardia* sp. ATCC202099)<br>NosH (*Streptomyces actuosus*)	54<br>49
perG/PerG	Actin_02406	1854/618	NocG (*Nocardia* sp. ATCC202099)<br>NosG (*Streptomyces actuosus*)	73<br>65
perF/PerF	Actin_02407	1398/466	NocF (*Nocardia* sp. ATCC202099)<br>NosF (*Streptomyces actuosus*)	47<br>44
perE/PerE	Actin_02408	2589/863	NocE (*Nocardia* sp. ATCC202099)<br>NosE (*Streptomyces actuosus*)	64<br>56
perD/PerD	Actin_02409	966/322	NocD (*Nocardia* sp. ATCC202099)<br>NosD (*Streptomyces actuosus*)	59<br>55
perC/PerC	Actin_02410	1221/407	NocC (*Nocardia* sp. ATCC202099)<br>NosC (*Streptomyces actuosus*)	81<br>71
perV/PerV	Actin_02411	1122/374	NocV (*Nocardia* sp. ATCC202099)	71
perR/PerR	Actin_02412	516/172	NocR (*Nocardia* sp. ATCC202099)	87
perQ/PerQ	Actin_02413	597/199	NocQ (*Nocardia* sp. ATCC202099)	78
perU/PerU	Actin_02414	1173/391	NocU (*Nocardia* sp. ATCC202099)	59
perT/PerT	Actin_02415	1122/374	NocT (*Nocardia* sp. ATCC202099)	59
perS1/PerS1	Actin_02416	1020/340	Oxidoreductase (*Streptomyces* sp.)<br>NDP-hexose-3-ketoreductase<br>(*Streptoalloteichus hindustanus*)	61<br>59
perP/PerP	Actin_02417	993/331	NocP (*Nocardia* sp. ATCC202099)<br>NosP (*Streptomyces actuosus*)	61<br>55
perB/PerB	Actin_02418	1185/395	NosB (*Streptomyces actuosus*)<br>NocB (*Nocardia* sp. ATCC202099)	55<br>54
perS2/PerS2	Actin_02419	858/286	UDP-glucose 4-epimerase<br>(*Amycolatopsis pretoriensis*)<br>SpeI (*Streptomyces spectabilis*)	44<br><br>43

Gene/Protein	Locus_tag	Length bp/aa	Similar Proteins	% aa Identity
perS3/PerS3	Actin_02420	819/273	macrocin O-methyltransferase (*Micromonospora viridifaciens*)<br>putative methyl transferase (*Streptomyces argenteolus*)	60<br>51
perS4/PerS4	Actin_02421	1137/379	rhamnosyltransferase (*Streptomyces* sp. SANK 60405)<br>glycosyltransferase (*Allokutzneria albata*)	40<br>39
perS5/PerS5	Actin_02422	786/262	class I SAM-dependent methyltransferase<br>(*Kitasatospora mediocidica*)<br>(*Micromonospora viridifaciens*)	53<br>51
perX/PerX	Actin_02423	1176/392	cytochrome P450<br>(*Actinobacteria bacterium* OK074)<br>(*Streptomyces gilvigriseus*)	41<br>40
perS6/PerS6	Actin_02424	1197/399	glycosyltransferase<br>(*Streptomyces aizunensis*)<br>(*Kutzneria* sp. 744)	45<br>41
perS7/PerS7	Actin_02425	1134/378	class I SAM-dependent methyltransferase<br>(*Actinoalloteichus hymeniacidonis*)<br>(*Nocardia puris*)	70<br>52
perS8/PerS8	Actin_02426	1044/348	glycosyltransferase<br>(*Streptomyces aizunensis*)<br>(*Streptomyces* sp. WMMB 714)	50<br>45
perS9/PerS9	Actin_02427	1086/362	glycosyltransferase<br>(*Amycolatopsis tolypomycina*)<br>(*Streptomyces* sp. PAN_FS17)	41<br>40
perS10/PerS10	Actin_02428	1404/468	NDP-hexose 2,3-dehydratase<br>(*Plantactinospora* sp. KBS50)<br>(*Amycolatopsis vancoresmycina*)	60<br>59
perS11/PerS11	Actin_02429	912/304	NDP-hexose 4-ketoreductase<br>(*Micromonospora* sp. CNZ309)<br>(*Micromonospora echinofusca*)	56<br>56
perS12/PerS12	Actin_02430	1305/435	NDP-hexose 3,4-dehydratase<br>(*Streptomyces minoensis*)<br>(*Streptomyces caniferus*)	71<br>70
perA/PerA	Actin_02431	435/145	NocA (*Nocardia* sp. ATCC202099)<br>NosA (*Streptomyces actuosus*)	59<br>67

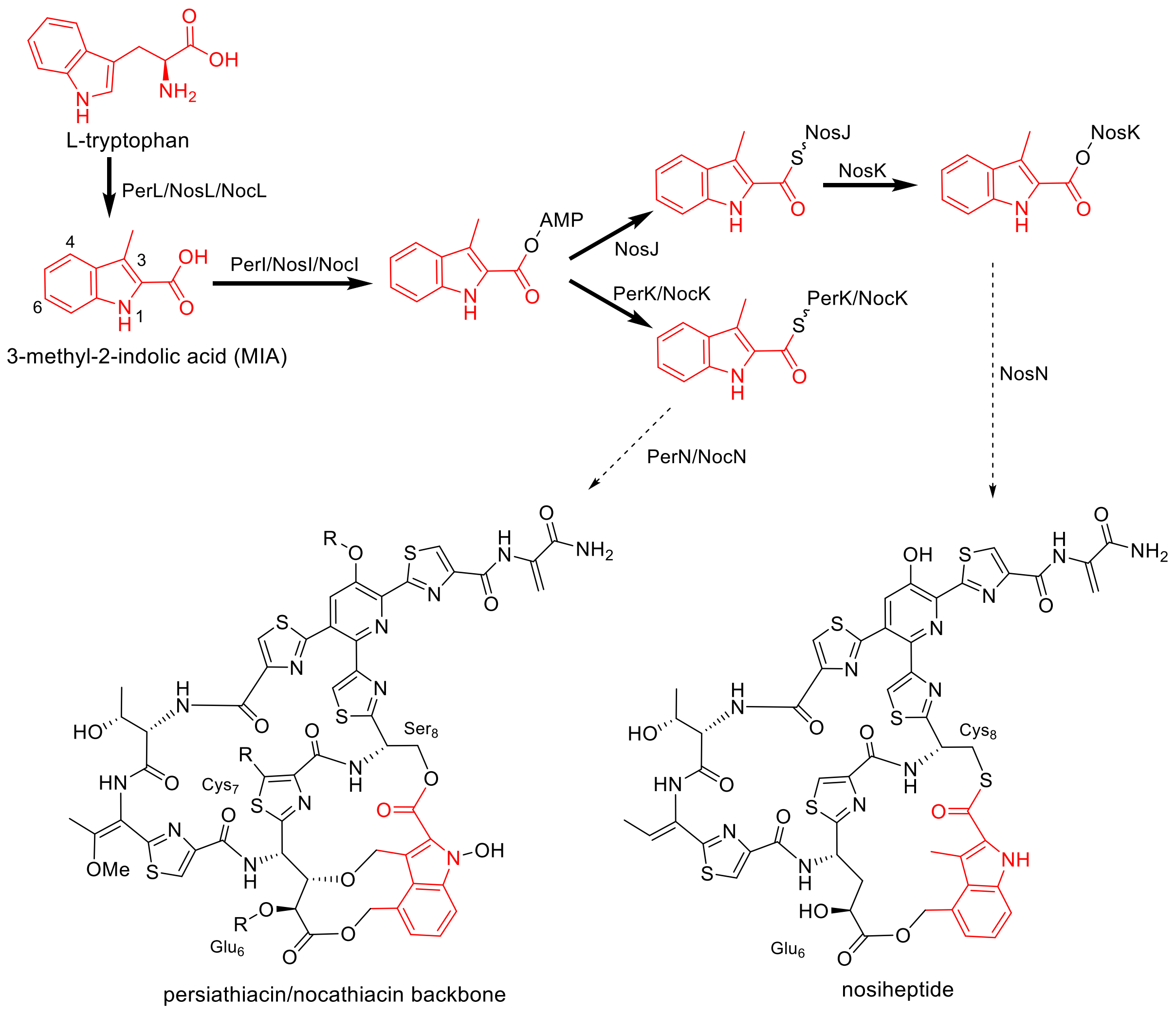

Figure S15. Similarities and differences in the activation and attachment of MIA to the processed core peptides of nosiheptide, persiathiacin, and nocathiacin (R-groups in persiathiacin and nocathiacin are shown in Figures 1 and 2 of the main manuscript).

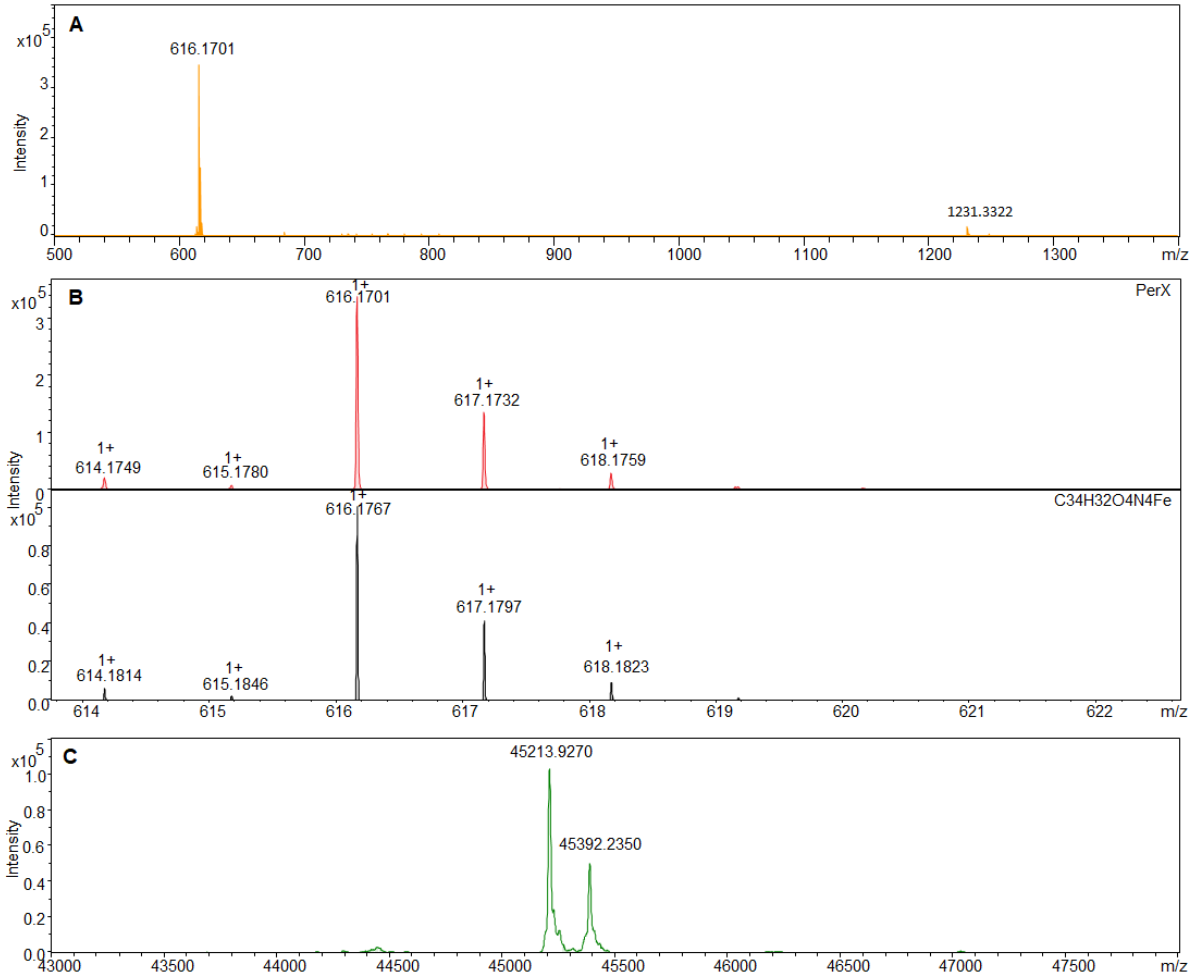

Figure S16. ESI-Q-TOF-MS analysis of purified recombinant PerX. (A) An ion with *m/z* = 616.1701, corresponding to $[M]^+$ for ferric haem, is observed. (B) Comparison of the measured (top) and simulated (bottom) spectra for the $[M]^+$ ion of ferric haem. (C) Deconvoluted mass spectrum of PerX. The measured masses correspond to the protein after loss of the N-terminal methionine residue (measured: 45213.92, calculated: 45215.35) and a glucuronidated derivative (measured: 45392.23, calculated: 45391.35).

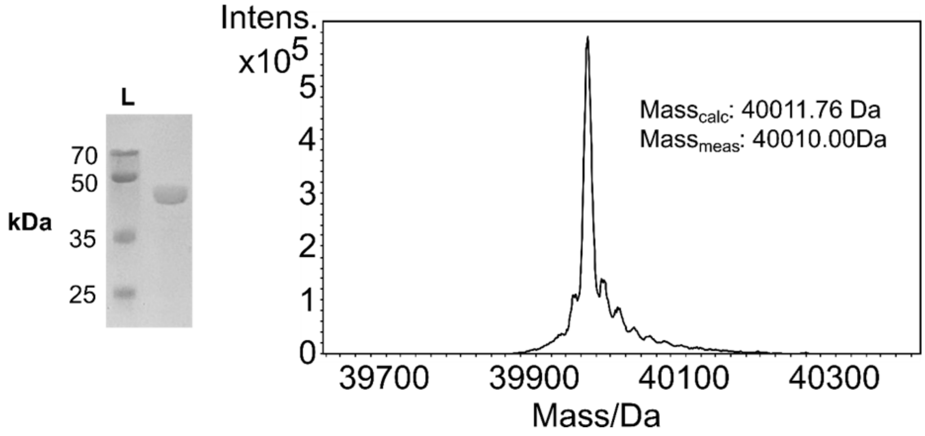

Figure S17. SDS-PAGE (left) and intact protein MS (right) of purified recombinant PerS4.

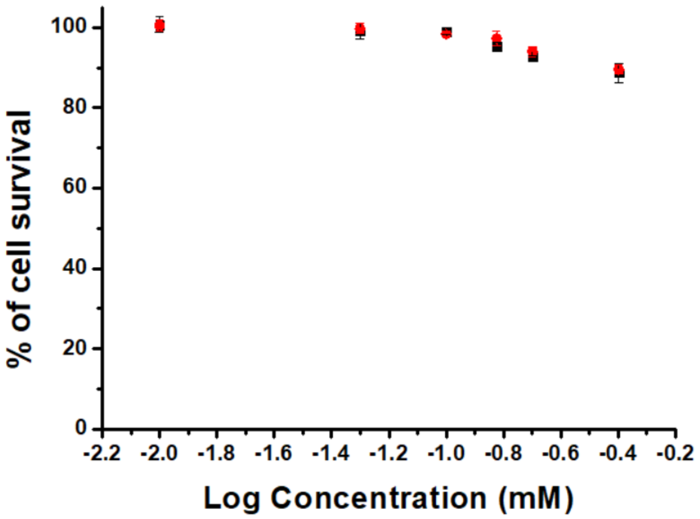

Figure S18. Percentage of A2780 ovarian cancer cells surviving after exposure to various concentrations of persiathiacin A ranging from 10 to 400 μM relative to untreated (vehicle) controls. These experiments included 24 h of drug exposure time and 72 h of recovery time in drug-free medium. Red circles and black squares represent data from two independent replicates.
